# Madagascar's grasses and grasslands: anthropogenic or natural?

**DOI:** 10.1098/rspb.2015.2262

**Published:** 2016-01-27

**Authors:** Maria S. Vorontsova, Guillaume Besnard, Félix Forest, Panagiota Malakasi, Justin Moat, W. Derek Clayton, Paweł Ficinski, George M. Savva, Olinirina P. Nanjarisoa, Jacqueline Razanatsoa, Fetra O. Randriatsara, John M. Kimeu, W. R. Quentin Luke, Canisius Kayombo, H. Peter Linder

**Affiliations:** 1 Comparative Plant and Fungal Biology, Royal Botanic Gardens, Kew, Richmond, Surrey TW9 3AB, UK; 2 Bioinformatics and Spatial Analysis, Royal Botanic Gardens, Kew, Richmond, Surrey TW9 3AB, UK; 3 CNRS-UPS-ENFA, UMR5174, EDB (Laboratoire Evolution et Diversité Biologique), Université Paul Sabatier, 118 route de Narbonne, 31062 Toulouse, France; 4 School of Geography, University of Nottingham, Nottingham NG7 2RD, UK; 5 School of Health Sciences, University of East Anglia, Norwich, Norfolk NR4 7TJ, UK; 6 Kew Madagascar Conservation Centre, II J 131 B, Ambodivoanjo, Ivandry, Antananarivo 101, Madagascar; 7 Département Botanique, Parc de Tsimbazaza, B.P. 4096, Antananarivo 101, Madagascar; 8 Ecole Supérieure des Sciences Agronomiques, Université d'Antananarivo, Antananarivo 101, Madagascar; 9 National Museums of Kenya, Museum Hill Road, PO Box 45166, Nairobi 00100, Kenya; 10 Forestry Training Institute, Olmotonyi, PO Box 943, Arusha, Tanzania; 11 Institute of Systematic Botany, University of Zurich, Zollikerstrasse 107, Zurich 8008, Switzerland

**Keywords:** Poaceae, neogene, endemism, species turnover, phylogenetic community assembly

## Abstract

Grasses, by their high productivity even under very low *p*CO_2_, their ability to survive repeated burning and to tolerate long dry seasons, have transformed the terrestrial biomes in the Neogene and Quaternary. The expansion of grasslands at the cost of biodiverse forest biomes in Madagascar is often postulated as a consequence of the Holocene settlement of the island by humans. However, we show that the Malagasy grass flora has many indications of being ancient with a long local evolutionary history, much predating the Holocene arrival of humans. First, the level of endemism in the Madagascar grass flora is well above the global average for large islands. Second, a survey of many of the more diverse areas indicates that there is a very high spatial and ecological turnover in the grass flora, indicating a high degree of niche specialization. We also find some evidence that there are both recently disturbed and natural stable grasslands: phylogenetic community assembly indicates that recently severely disturbed grasslands are phylogenetically clustered, whereas more undisturbed grasslands tend to be phylogenetically more evenly distributed. From this evidence, it is likely that grass communities existed in Madagascar long before human arrival and so were determined by climate, natural grazing and other natural factors. Humans introduced zebu cattle farming and increased fire frequency, and may have triggered an expansion of the grasslands. Grasses probably played the same role in the modification of the Malagasy environments as elsewhere in the tropics.

## 1. Background

Grasses have transformed the planet. Since the Oligocene, the expanding dominance of grasses has led to a reduction in forested vegetation, has transformed the herbivore faunas and their associated predators and has dramatically increased the frequency of fire. Grasses have acted as biotic modifiers, generating a whole new set of ecosystems and selective environments that have led to the reduction or demise of some older forms, but stimulated the evolution of newer forms. Consequently, knowing the origins of grasslands in any region is central to the interpretation of the Neogene environments in that region [1,2].

Grasses have a set of traits that have allowed them to expand their habitats and become dominant in many biomes [1]. These include C_4_ photosynthesis that allows them to remain highly productive under low *p*CO_2_, underground buds that allow the plants to survive fires and intense grazing, silica bodies that may limit grazing damage, fast-growing foliage that can rapidly generate new biomass to replace material removed by fires or grazing, and seeds with well-developed embryos that allow the plants to rapidly invade potentially suitable habitat. Another set of traits have been linked to frost tolerance, and these have allowed the family to expand dramatically into the colder high-latitude regions, building steppe grasslands (e.g. [1]).

The fossil record documents the Miocene expansion of grasslands, both from the presence of phytoliths in Turkey [3] and North America [4], and from the evolution of hypsodont grazer teeth in North America [2,5,6], although these studies may not be directly applicable to the humid tropics. The development of carbon isotope analysis, from palaeosols, bones [7] and from plant leaf waxes [8], has led to the recent realization that these early grasslands were C_3_ dominated, and that they were transformed to C_4_ grasslands only in the Late Miocene–Pliocene [2,8,9]. Often the spread of C_4_ grasses is associated with an increase in fire, as evidenced from the increase of charcoal in the deposits [8].

Grasslands (including wooded grassland, tapia and palm savannah) are extensive in Madagascar, covering at least 65% of the island not including cultivation [10]. There has been an ongoing debate about the age and consequences of the establishment of the Malagasy grasslands. Early botanists (e.g. Perrier de la Bâthie [11], Humbert [12], Koechlin [13]) argued that all Malagasy grasslands are secondary and the result of the anthropogenic introduction of fire and zebu cattle. Consequently, this modification is seen as being post human settlement (2000–4500 BP [14,15]). Bond *et al*. [16] take the opposite point of view, suggesting that extensive grasslands have existed before the arrival of humans, and that consequently humans had a lesser effect on the expansion of grasslands. Other intermediate scenarios have also been presented but without detail or concrete evidence. Stable isotope data from northwest Madagascar indicate a massive increase in C_4_ grass in the past millennium, subsequent to the first human settlements. However, traces of C_4_ isotopes indicate that there were C_4_ grassland patches before the first human expansion [17]. Population genetic data of the golden-crowned sifaka (*Propithecus tattersalli*), a forest-dwelling lemur in northern Madagascar, suggest that population contractions, presumably due to the forests being replaced by grassland, preceded the arrival of humans, and may have been driven by climatic changes [18]. Palaeopalynological and macrofossil data from central and southwestern Madagascar also indicate a major vegetation transformation prior to the arrival of humans, probably in response to climate changes [19–22]. Evolutionary radiations restricted to open areas have been documented in both ants [23] and sedges [24] but there have been no similar studies of broader taxonomic groups. Consequently, there is no dominant narrative on the evolutionary history of the Malagasy grasses and grasslands.

Here we contribute new evidence to the debate on the origin and evolutionary history of the Malagasy grass flora and grassland. We first address the question of whether the grass flora is natural in Madagascar, and diversified *in situ*, or whether it is a recently introduced flora that spread into anthropogenically disturbed habitats. Then we explore the ecology of the grass flora, and in particular we ask whether the flora of each ecoregion in Madagascar is distinct, or whether there is a single grass flora across the whole island. We test whether the locally distinct grass floras are the result of filtering a larger, widespread flora or due to local evolution. Finally, we test whether the Malagasy grass flora evolved in disturbed habitats, or whether at least some species are not adapted to fire, grazing and cultivation, indicative of an evolution under low disturbance regimes.

**Figure 1.**
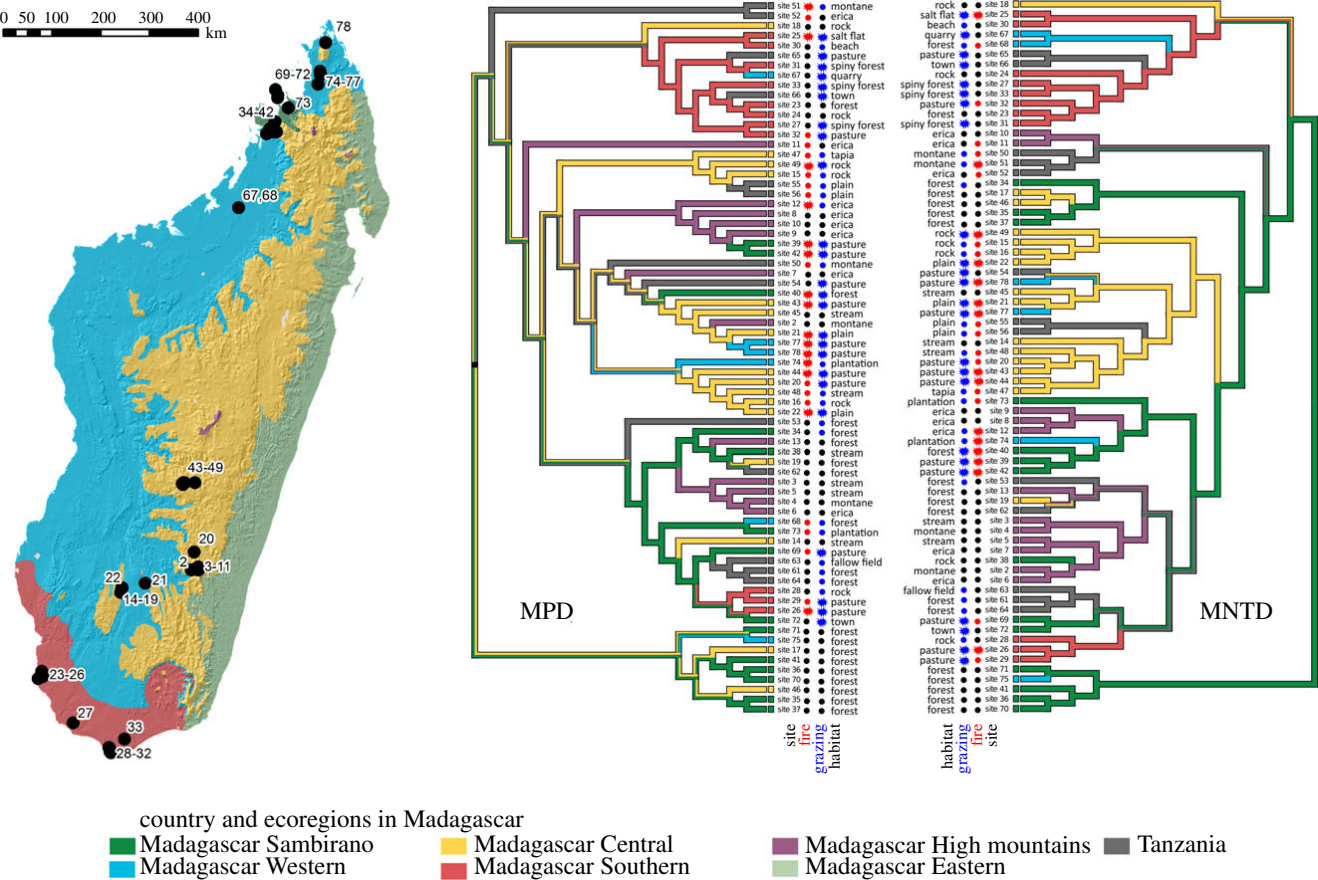
Study sites in Madagascar and phylogenetic beta diversity based on mean pairwise distance (MPD) and mean nearest taxon distance (MNTD). Ecoregions follow [27] and are marked in colour; habitat is marked in text. Fire intensity and physical disturbance intensity are marked with symbols: black circles indicate no disturbance; small red and blue circles indicate intermediate levels of fire and physical disturbance, respectively; big red and blue stars indicate high levels of fire and physical disturbance, respectively. All four traits have significant phylogenetic structure (posterior tail probability, *p* < 0.01). Grasslands are mostly distributed in the Western (52.5%) and Central (38.1%) parts, but are also present in other regions, in Southern (4.8%), Eastern (3.9%), Sambirano (0.6%) and High mountains ecoregion (0.2%). Note that the Eastern ecoregion was not sampled at all.

In order to address these questions, we conducted a critical taxonomic review of the Malagasy grass flora, updating the earlier work of Bosser [25] and E. J. Judziewicz (2009, unpublished data, except for [26]), resulting in an updated checklist of the grass flora (electronic supplementary material, S5) and more precise estimates of its endemism. In order to assess whether the grass communities show signs of expansion or sensitivity to disturbance, we sampled plots in five major ecoregions [27–29] of the island (figure 1). We built a phylogeny including all sampled species, and transformed this into an ultrametric tree using the penalized likelihood criterion [30]. We used this to calculate the patterns of phylogenetic community assembly and to detect signals of phylogenetic filtering in the assemblage of variously disturbed grassland communities.

## 2. Material and methods

### (a) Global endemism in Poaceae

**Figure 2.**
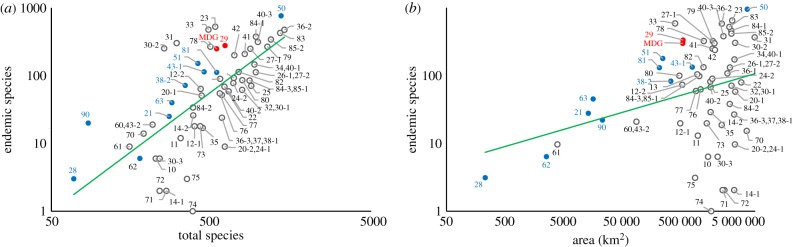
Endemism in Malagasy grass flora compared to island (blue) and continental (grey) regions of the world. Madagascar (red) is included twice, as a separate island (MDG) and again together with the surrounding islands (29), to demonstrate that endemism is similar in both cases. Numbers indicate regions of the world following the Taxonomic Database Working Group, which are listed in the electronic supplementary material, table S1. (*a*) Number of Poaceae endemics plotted against species richness (data from GrassBase [31]); *R*^2^ = 0.47. Poaceae endemicity in the Malagasy floristic region is in the line with other subtropical islands. (*b*) Poaceae endemism plotted against land area; *R*^2^ = 0.09. Madagascar shows high endemism for its land area, comparable to New Zealand.

Species numbers and distribution data on all 11 313 accepted species of Poaceae at the Taxonomic Databases Working Group (TDWG) level 3 were extracted from GrassBase [31], described in [32], and filtered into total richness and total endemic species numbers. Percentage endemism was calculated for each TDWG level 3 area (figure 2). Areas were obtained from TDWG shapefiles and [33] http://www.kew.org/science-conservation/research-data/resources/gis-unit/tdwg-world in QGIS [34] using the Eckert VI projection (http://bdtracker.cybertaxonomy.africamuseum.be/node/641).

### (b) Grasslands in Madagascar

Ground-truthed models of Madagascar's vegetation included two types of grassland: plateau grassland–wooded grassland mosaic, and wooded grassland–bushland [10]. These are primarily on basement rocks (*ca* 56%) followed by sandstone (*ca* 15%) and may include components of secondary vegetation due to limitations of the original vegetation mapping. ARCGIS 10.1 [35] was used to quantify known grassland in each of Humbert's ecoregions [27] (figure 1).

### (c) Poaceae diversity in Madagascar and Tanzania

A long-term taxonomic review of all Malagasy Poaceae was carried out to build a checklist of 541 species including 216 endemic species (electronic supplementary material, S4). Herbarium specimens were studied at K, P and TAN herbaria (http://sciweb.nybg.org/science2/IndexHerbariorum.asp) concurrently with a fieldwork programme and a literature survey. Detailed revisions of several groups are published separately [36–39]. Distribution ranges were defined for each species collected for this study, using herbarium specimens to record the presence/absence in the six Malagasy ecoregions: Central, Eastern, High Mountain, Sambirano, Southern and Western [27]. In cases of uncertainty, we followed a conservative approach by assuming a broader distribution. Tanzania's grass flora was chosen to represent tropical continental grass floras to compare with Madagascar because it has the most similar climate, vegetation and land area, it is geographically adjacent to Madagascar, and its grasses and grasslands are well documented.

### (d) Field sampling and quantifying disturbance

The grass flora was sampled at 60 sites in Madagascar, from five ecoregions, representing much of the climate range and the whole altitude range. Only the eastern rainforest region was not sampled. We selected sites to represent the widest range of habitats, and to include both undisturbed and highly disturbed locations (figure 1; electronic supplementary material, S2 and figure S3). Thirteen Afromontane and mid-elevation open grassland sites in Tanzania were also studied (electronic supplementary material, S2). Each site was placed in a visually uniform vegetation community. Plots were placed along four transects and all Poaceae species were listed and collected. An associated vegetation list, soil, geomorphology and disturbance data were recorded; sampling methodology is described in the electronic supplementary material, S1 and illustrated in figure S2. Species identification for fertile material was carried out at K, P and TAN; sterile material identification was carried out using plastid *rbcL* sequences. Two hundred and six species were recorded in total (electronic supplementary material, S2 and S3). Sites with no recorded burning within the past 10 years, no or very occasional grazing and no natural disturbance were assigned as human impact score 0. Sites burned within the past 10 years, with limited human use or with natural disturbance such as streams or frequent storm damage were assigned as human impact score 0.5. Heavily used pastures or communal land were assigned human impact score 1. Multiple regression analyses estimating the independent effects of fire and physical disturbance on mean pairwise distance (MPD) and mean nearest taxon distance (MNTD) were carried out using [40] and adjusted for altitude and spatial autocorrelation using the user-contributed spreg package for Stata and Stata v. 12.

### (e) Phylogeny and phylogenetic diversity

The plastid regions *rbcL*, *ndhF* and *matK* were sequenced for each species following the methodology described in [41]. A phylogenetic tree of all species found in the 73 sites was produced using the maximum-likelihood criteria as implemented in the program RAxML v. 8.1.11 [42] and performed on the Cipres Science Gateway portal (https://www.phylo.org) with a rapid bootstrapping approach (1000 replicates). The tree was made ultrametric using the penalized likelihood criteria [30] as implemented in the function *chronos* and the model *correlated* of the R package ape [43–45] and assigning the value of 1.0 to the crown node of the tree (electronic supplementary material, S3). The package picante [46] was used to calculate MPD and MNTD [47]. The observed phylogenetic relatedness was compared to the expected pattern using the functions *ses.mpd* and *ses.mntd* with the ‘richness’ null model, taking into account species abundances in each plot, and using 9999 randomizations.

Phylogenetic beta diversity was calculated using the function *comdistnt*, the ‘among-community equivalent of MPD and MNTD’ [46] and taking into account species abundance in each plot. Communities were clustered based on their phylogenetic relatedness using the hierarchical cluster analysis implemented in the package *stats* [44] under the function *hclust*. Phylogenetic structure was assessed by comparing the trees to 1000 randomly generated trees within the Mesquite system for phylogenetic computing [48].

## 3. Results and discussion

### (a) The grass flora: global endemism

Theory predicts that if the grasses have had a long evolutionary history in Madagascar, then the levels of endemism should be high, and comparable to the levels of endemism observed in other large, subtropical islands. If, however, grasses were recently introduced into Madagascar, or only recently had sufficient habitat to expand into, then the levels of endemism should be much lower than in environmentally comparable islands. We find that 217 of 541 grass species (electronic supplementary material, S4), or 40%, are endemic to the island. Furthermore, 11 of 140 genera are endemic [49,50]. Madagascar has more endemic grass species in proportion to the total grass flora than most other regions (figure 2*a*). The highest proportions of grass endemics are recorded for the central Asian regions (Caucasus to southern Siberia) as well as the Antarctic islands. As predicted for an older grass flora, the proportion of grass endemics in Madagascar is comparable to that found for Australia, southeastern North America and New Zealand: thus subtropical, and in part island, floras.

Furthermore, Madagascar has a high level of grass endemism compared with its surface area (figure 2*b*). As expected, the per cent endemism relative to the area is low for countries and regions that contain part of large deserts like the tundra, the Sahara or the Kalahari (e.g. Arabia, Canada, West Tropical Africa, West Asia, South Tropical Africa, North Africa and North-central Africa). A more or less average per cent endemism is observed for Australia, India, South America, Malesia, Southern Africa and the USA. A high level of endemism relative to the surface area is shown for islands and archipelagos like New Zealand, Japan, Papuasia, as well as equatorial continental areas such as Brazil. Madagascar groups comfortably with these tropical and subtropical regions and islands.

These results indicate that, although at 40% endemism the grass flora has less than half the level of endemism recorded for the angiosperm flora in general [51], this is high compared with the levels of endemism in the grasses globally. This level of endemism is consistent with the hypothesis that the Malagasy evolutionary history of the grass flora has been as long as that of the other major islands, and is certainly much older than human settlement on the island.

### (b) The grass flora: composition comparison with East Africa

Island floras are often unbalanced, with a very different distribution of species diversity among higher taxa from the adjacent mainland [52]. This imbalance is presumed to be the result of low immigration rates, resulting in few lineages radiating into a large diversity of habitats. No such imbalance is evident in the Malagasy Poaceae. The proportions of species in the most important subfamilies and tribes closely reflect that of East Africa with its famous natural savannahs (electronic supplementary material, figure S1), except for the Bambusoideae, which radiated in the wet eastern forests of Madagascar. This suggests that there is a close connection with the African grass flora, as demonstrated for the majority of Malagasy flora by Buerki *et al*. [53]. Niche conservatism [54] suggests that the grassland grass flora is adapted to similar environments to the East African grasslands, noted for its regular and intense grazing regime and frequent fires.

### (c) Regionalism in the grass communities

If the grass flora evolved in one ecoregion of Madagascar and then expanded with the arrival of humans over the whole island, then the degree of regionalism in the flora should be very low (or even absent), and local endemism should be restricted to the area where the flora evolved, whereas the newly occupied regions should simply have subsets of the refugial flora.

We tested for regionalization in the Malagasy grass flora by calculating the phylogenetic beta diversity (pß) [55] among the sampled sites, and using this matrix to cluster the sites. This approach uses the phylogenetic information, so clustering together sites that have closely related species, even if there is allopatric replacement in the species. It is preferred to clustering on shared species presences, which cannot group sites with closely related but different species [56,57]. The results (figure 1) show that sites from the same ecoregion are significantly clustered together, irrespective whether pß is calculated using MPD or MNTD [47]. MPD is more sensitive to deep phylogenetic differences than MNTD. Both geographical proximity (belonging to the same ecoregion) and habitat similarity (belonging to the same vegetation type) impact on the relatedness among the sites, and this is probably the reason why neither fits perfectly on the diagram of how related the sites are. The significant spatial and ecological regionalization in the Malagasy grass flora is not consistent with the grasslands spreading from a single grassy biome, but more with a long-term grass component in each of the biomes, evolving specialization to these habitats. Ecologically such regionalization is not surprising, considering the remarkably steep environmental gradients within Madagascar, in terms of average temperature (altitudinal gradient), in total rainfall (east to west) and in the length of the dry season (southwest to northeast) [10].

### (d) Regional endemism

However, it remains possible that the regionalization is the result of grass species, which may have evolved on other continents, being filtered into these ecologically diverse habitats, rather than having evolved *in situ*. If they evolved *in situ*, then there should be a high degree of regional endemism. We found a surprisingly high degree of regional endemism. Out of 57 Malagasy endemic species (of 206 species total in our study) found in the 76 sites, 33 are restricted to a single ecoregion (i.e. narrow endemics) and 14 are restricted to two ecoregions, and only 10 species (18%) are recorded from three or more ecoregions. Endemism ranges from zero (Isalo forest, Horombe plateau grassland and Tsimananpetsotsa salt flats) to 100% (Andringitra plateau, Isalo rocks and Manongarivo forest, S2). The level of endemism is significantly higher in Malagasy than in Tanzanian sites (table 1). There are also significant differences among the Malagasy ecoregions, with the lowest level of endemism found in the Plateau ecoregion, and the highest levels in the more extreme habitats: at high altitude and in the southern spiny forests. All four published comprehensive local Malagasy grassland surveys [58–61], and two complete Tanzanian grassland surveys [62,63] revealed a similar pattern of high Madagascan local endemism, compared with virtually none in Tanzania.

**Table 1.** Endemicity of grass species recorded in this study, in comparison to published checklists. 9–61% of the species in every ecoregion of Madagascar are single region endemics, and 21–94% are endemic to Madagascar. Endemicity in Tanzania is massively lower with 0–2% of species endemic to Tanzania and 2–9% restricted to three countries including Tanzania.

Madagascar	total number of Poaceae species	endemic species restricted to Madagascar (% total)	narrow endemic species restricted to a single ecoregion in Madagascar (% total)
Central ecoregion, 16 sites in this study	60	27 (45)	10 (17)
Central ecoregion, Itremo Protected Area [58]	100	35 (35)	14 (14)
Central ecoregion, southwestern savannahs [59]	43	9 (21)	4 (9)
High Mountains ecoregion, 12 sites in this study	33	19 (58)	11 (33)
High Mountains ecoregion, Andringitra National Park [60]	18	17 (94)	11 (61)
Sambirano ecoregion, 14 sites in this study	33	12 (36)	5 (15)
Sambirano ecoregion, Manongarivo Reserve [61]	42	23 (55)	11 (26)
Southern ecoregion, 11 sites in this study	27	14 (52)	12 (44)
Western ecoregion, 7 sites in this study	22	7 (32)	4 (18)
Madagascar total, 60 sites in this study	145	70 (48)	42 (29)

Tanzania	total number of Poaceae species	endemic species restricted to three African countries or fewer (% total)	narrow endemic species restricted to Tanzania (% total)
Tanzania total, 13 sites in this study	65	6 (9)	1 (2)
Tanzania, Mkomazi National Park [62]	123	3 (2)	0
Tanzania, Selous Game Reserve [63]	239	12 (5)	1 (0.5)

### (e) Response to disturbance

Communities exposed to disturbance regimes under which they did not evolve should show more phylogenetic clustering than communities with disturbance regimes under which they have evolved [64–66]. Cattle grazing may constitute a new disturbance regime in Madagascar. There is no evidence of native Malagasy ungulates or any animals similar to the African savannah grazers [67]. The main source of information on the diets of extinct herbivores is carbon isotope data: the pygmy hippos ate a high proportion of C_4_ plants [68], the elephant birds consumed primarily C_3_ [69], while the two species of giant tortoises differed in their preferences [70]. While C_4_ isotopes in the diet indicate open habitats, C_3_ diets could derive from either a C_3_-dominated grassland or from a eudicot diet [9]. Comparison to extant relatives is the only other source of available evidence: this indicates that giant tortoises were probably the most influential past grazers of open habitats [71]. The probable giant lemur diet of leaves, fruits and seeds has also been inferred by comparison to extant relatives [72]. Fire is accepted as a natural part of Madagascar's ecosystems from at least 10 000 BP and likely as long as 120 000 BP [19–22].

We expect that disturbed grassland will be phylogenetically clustered relative to undisturbed grassland. In order to test this, we calculated MPD and MNTD for all sites. We assigned study sites as having low, medium or high levels of physical disturbance (grazing and trampling) and of fire, and tested whether these differ for MPD and MNTD using (i) one-way analysis of variance comparing each measure across groups defined by each level of disturbance and (ii) multiple regression analysis including the effects of both physical disturbance and fire, adjusting for altitude and any spatial autocorrelation between sites.

**Figure 3.**
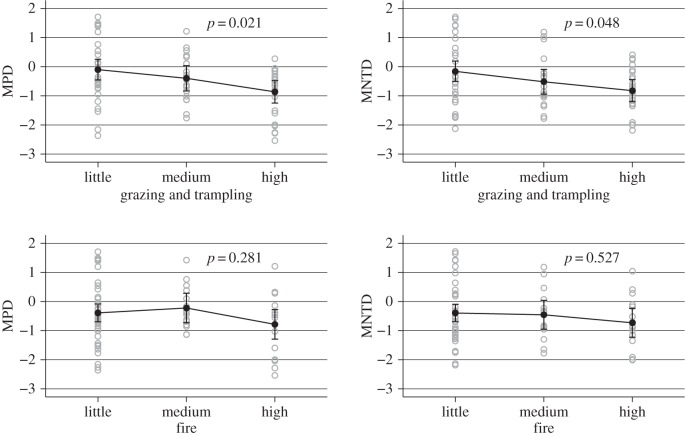
MPD and MNTD compared between three levels of disturbance, for physical disturbance and for disturbance by fire. *P*-values correspond to one-way ANOVA across groups. Physical disturbance is associated with significantly lower MPD and MNTD while fire has no significant effect. Open circles correspond to values for individual sites, filled circles are group means with 95% confidence intervals.

One-way ANOVA shows that physical disturbance including grazing and trampling is associated with significantly lower MPD and MNTD, while fire has no effect (figure 3). This is confirmed by the multiple regression analyses for each outcome, which show no independent effect of fire but endemicity scores almost 1s.d. lower in areas that have high levels of physical disturbance. These findings are consistent with the postulate that the grasslands, on the whole, have not been exposed to heavy grazing, but evolved under conditions of light grazing or no grazing. Fire may have already been present as these ecosystems emerged.

Finally, we tested whether disturbed grasslands in Madagascar are derived from the local grasslands (possibly by filtering out those species incapable of tolerating the disturbance regimes), or whether they are assembled from a set of introduced, disturbance-tolerant taxa, by investigating whether these sites group together. We found that they did not (figure 1), and that we could not reject the null hypothesis that they are random in the cluster analysis. This indicates that the disturbed grasslands are assembled by filtering species from native grasslands.

## 4. Conclusion

The remarkably high levels of endemism constitute compelling evidence that the Malagasy grass flora is ancient, and the very similar proportional representation of the subfamilies to that found in East Africa suggests that it was probably assembled with much dispersal to and from Africa. We find no evidence that suggests that the flora is any younger than that of Africa.

We also established that the grassland communities are most likely Neogene in age, and not the result of Holocene human impacts. This is supported by the strong regionalization in the grass flora (indicating a long evolutionary history over the whole island), and a very high level of local endemism. The latter suggests a high level of niche or habitat specialization in the grass communities.

Finally, we show that the grasslands most likely evolved under a less intensive physical disturbance regime than what they are currently experiencing. This is inferred from the significant effect of grazing and trampling on the phylogenetic assembly of these communities, indicating that not all local species can tolerate the modern disturbance regime.

Although our research suggests that grassland formations and indeed, grasses, on Madagascar are natural, we did not establish what the relative impacts of climate change and human disturbance may have been on the balance between forest and grassland in Madagascar. It is possible that human disturbance may have resulted in a much larger modern extent of grassland than in pre-human settlement Madagascar. The balance between grassland and forest in Madagascar may be dynamic, and responding both to climate changes (e.g. the Mid-Holocene drought) and human-mediated disturbances such as introduced zebu cattle and more frequent fires. These results also argue for the need to conserve and protect species-rich and highly endemic grasslands, and that it is time to consider such habitats as being part of the biologically unique heritage of Madagascar.

## Supplementary Material

ESM Figures and Tables

## Data accessibility

Data on global endemism of the Poaceae are available for download as part of GrassBase at http://www.kew.org/data/grasses-syn/download.htm and TDWG shapefiles are available at http://www.kew.org/science-conservation/research-data/resources/gis-unit/tdwg-world. DNA sequences have been deposited at the European Nucleotide Archive with the following numbers: LN906638–LN906796 (*matK*), LN907872–LN908034 (*rbcL*) and LN908035–LN908194 (*ndhF*). Phylogenetic data are available under TreeBase ID 18532.

## References

[1] Kellogg EA 2015 Flowering Plants. Monocots: Poaceae. In The families and genera of vascular plants series (ed. Kubitzki K), vol. 13 Heidelberg, Germany: Springer.

[2] Strömberg CAE 2011 Evolution of grasses and grassland ecosystems. Annu. Rev. Earth Planet. Sci. 39, 517–544. (10.1146/annurev-earth-040809-152402)

[3] Strömberg CAE, Werdelin L, Friis EM, Sarac G 2007 The spread of grass-dominated habitats in Turkey and surrounding areas during the Cenozoic: phytolith evidence. Palaeogeogr. Palaeoclimatol. Palaeoecol. 250, 18–49. (10.1016/j.palaeo.2007.02.012)

[4] Strömberg CAE 2005 Decoupled taxonomic radiation and ecological expansion of open-habitat grasses in the Cenozoic of North America. Proc. Natl Acad. Sci. USA 102, 11 980–11 984. (10.1073/pnas.0505700102) PMC1189350 16099827

[5] Strömberg CA, Dunn RE, Madden RH, Kohn MJ, Carlini AA 2013 Decoupling the spread of grasslands from the evolution of grazer-type herbivores in South America. Nat. Commun. 4, 1478 (10.1038/ncomms2508) 23403579

[6] Strömberg C 2006 Evolution of hypsodonty in equids: testing a hypothesis of adaptation. Paleobiology 32, 236–258. (10.1666/0094-8373(2006)32%5B236:EOHIET%5D2.0.CO;2)

[7] Cerling TE, Wang Y, Quade J 1993 Expansion of C4 ecosystems as an indicator of global ecological change in the late Miocene. Nature 361, 344–345. (10.1038/361344a0).

[8] Hoetzel S, Dupont L, Schefuss E, Rommerskirchen F, Wefer G 2013 The role of fire in Miocene to Pliocene C_4_ grassland and ecosystem evolution. Nat. Geosci. 6, 1027–1030. (10.1038/ngeo1984).

[9] Feakins SJ, Levin NE, Liddy HM, Sieracki A, Eglinton TI, Bonnefille R 2013 Northeast African vegetation change over 12 m.y. Geology 41, 295–298. (10.1130/G33845.1).

[10] Moat J, Smith P 2007 Atlas of the vegetation of Madagascar. Kew, London, UK: Royal Botanic Gardens.

[11] Perrier de la Bâthie H 1921 La végétation malgache. Annales du Musée colonial de Marseille, 3me ser. 9, 1–266.

[12] Humbert H 1927 Destruction d'une flore insulaire par le feu: principaux aspects de la végétation à Madagascar. Mèm. Acad. Malgache 5, 1–80.

[13] Koechlin J, Guillaumet J-L, Morat P 1974 Flore et Végétation de Madagascar. Vaduz: Gantner.

[14] Dewar RE, Radimilahy C, Wright HT, Jacobs Z, Kelly GO, Berna F 2013 Stone tools and foraging in northern Madagascar challenge Holocene extinction models. Proc. Natl Acad. Sci. USA 110, 12 583–12 588. (10.1073/pnas.1306100110) PMC3732966 23858456

[15] Gommery D, Ramanivosoa B, Faure M, Guérin C, Patrice KH, Sénégas F, Randrianantenaina H 2011 Les plus anciennes traces d'activités anthropiques de Madagascar sur des ossements d'hippopotames subfossiles d'Anjohibe (Province de Mahajanga). C R Palevol. 10, 271–278. (10.1016/j.crpv.2011.01.006)

[16] Bond WJ, Silander JA, Ranaivonasy J, Ratsirarson J 2008 The antiquity of Madagascar's grasslands and the rise of C_4_ grassy biomes. J. Biogeogr. 35, 1743–1758. (10.1111/j.1365-2699.2008.01923.x)

[17] Crowley BE, Samonds KE 2013 Stable carbon isotope values confirm a recent increase in grasslands in northwestern Madagascar. Holocene 23, 1066–1073. (10.1177/0959683613484675)

[18] Quéméré E, Amelot X, Pierson J, Crouau-Roy B, Chikhi L 2012 Genetic data suggest a natural prehuman origin of open habitats in northern Madagascar and question the deforestation narrative in this region. Proc. Natl Acad. Sci. USA 109, 13 028–13 033. (10.1073/pnas.1200153109). PMC3420155 22826244

[19] Burney DA 1987 Late quaternary stratigraphic charcoal records from Madagascar. Quat. Res. 28, 274–280. (10.1016/0033-5894(87)90065-2)

[20] Burney DA 1987 Late Holocene vegetational change in Central Madagascar. Quat. Res. 28, 130–143. (10.1016/0033-5894(87)90038-X)

[21] Gasse F, Van Campo E 2001 Late Quaternary environmental changes from a pollen and diatom record in the southern tropics (Lake Tritrivakely, Madagascar). Palaeogeogr. Palaeoclimatol. Palaeoecol. 167, 287–308. (10.1016/S0031-0182(00)00242-X)

[22] Burney DA 1993 Late Holocene environmental changes in arid Southwestern Madagascar. Quat. Res. 40, 98–106. (10.1006/qres.1993.1060)

[23] Fisher BL, Robertson HG 2002 Comparison and origin of forest and grassland ant assemblages in the High Plateau of Madagascar (Hymenoptera: Formicidae) 1. Biotropica 34, 155–167. (10.1111/j.1744-7429.2002.tb00251.x)

[24] Muasya AM, Larridon I, Reynders M, Huygh W, Goetghebeur P, Cable S, Simpson DA, Gehrke B 2013 The Cyperaceae in Madagascar show increased species richness in upland forest and wetland habitats. Scr. Botanica Belgica 50, 238–243.

[25] Bosser J 1969 Graminées des paturages et des cultures a Madagascar. Paris, France: Orstom.

[26] Judziewicz EJ 2009 *Toliara* (Poaceae, Chloridoideae, Cynodonteae), a new grass genus endemic to southern Madagascar. Adansonia 31, 273–277. (10.5252/A2009n2a4)

[27] Humbert H 1955 Les Territoires Phytogéographiques de Madagascar. Leur Cartographie. Colloque sur les Régions Ecologiques du Globe, Paris 1954. Ann. Biol. 31, 195–204.

[28] Faramalala MH 1995 Formations Végétales et Domaine Forestier National de Madagascar. Conservation International (et al.), 1 map.

[29] Faramalala MH 1988 Etude de la Végétation de Madagascar à l'aide des Données Spaciales. Toulouse, France: Université Paul Sabatier.

[30] Sanderson MJ 2002 Estimating absolute rates of molecular evolution and divergence times: a penalized likelihood approach. Mol. Biol. Evol. 19, 101–109. (10.1093/oxfordjournals.molbev.a003974) 11752195

[31] Clayton WD, Vorontsova MS, Harman KT, Williamson H 2015 GrassBase—The Online World Grass Flora. Kew: Royal Botanic Gardens Kew.

[32] Vorontsova MS, Clayton WD, Simon BK 2015 Grassroots e-floras in the Poaceae: growing GrassBase and GrassWorld. PhytoKeys 48, 73–84. (10.3897/phytokeys.48.7159). PMC4408733 25941449

[33] Brummitt RK, Pando F, Hollis S, Brummitt NA 2001 World geographical scheme for recording plant distributions, 2 edn Carnegie Mellon University, Pittsburgh: International Working Group on Taxonomic Databases For Plant Sciences, Hunt Institute for Botanical Documentation.

[34] Quantum GIS Development Team 2015 Quantum GIS Geographic Information System. Open Source Geospatial Foundation Project. See http://qgis.osgeo.org.

[35] ESRI. 2012 ArcGIS. Redlands, CA: ESRI.

[36] Vorontsova MS, Ratovonirina G, Randriamboavonjy T 2013 Revision of *Andropogon* and *Diectomis* (Poaceae: Sacchareae) in Madagascar and the new *Andropogon* *itremoensis* from the Itremo Massif. Kew Bull. 68, 193–207. (10.1007/S12225-013-9443-3).

[37] Vorontsova MS 2014 Two new species of *Panicum* sensu lato (Poaceae: Panicoideae) from Madagascar. Kew Bull. 69 (2)-9511:1–7 (10.1007/s12225-014-9511-3)

[38] Vorontsova MS, Nanjarisoa OP, Besnard G 2014 Three new grass records for Madagascar. Candollea 69, 85–87. (10.15553/c2014v691a10)

[39] Vorontsova MS, Haevermans T, Haevermans A, Razanatsoa J, Besnard G 2015 The genus *Sartidia* (Poaceae: Aristidoideae) in Madagascar. Syst. Bot. 40, 448–453. (10.1600/036364415X688367)

[40] Drukker DM, Prucha IR, Raciborski R 2011 Maximum-likelihood and generalized spatial two-stage least-squares estimators for a spatial-autoregressive model with spatial-autoregressive disturbances. University of Maryland, Department of Economics.

[41] Besnard G, Christin PA, Male PJ, Lhuillier E, Lauzeral C, Coissac E, Vorontsova MS 2014 From museums to genomics: old herbarium specimens shed light on a C_3_ to C_4_ transition. J. Exp. Bot. 65, 6711–6721. (10.1093/jxb/eru395) 25258360

[42] Stamatakis A 2014 RAxML Version 8: a tool for phylogenetic analysis and post-analysis of large phylogenies. Bioinformatics 30, 1312–1313. (10.1093/bioinformatics/btu033) 24451623 PMC3998144

[43] Paradis E, Claude J, Strimmer K 2004 APE: analyses of phylogenetics and evolution in R language. Bioinformatics 20, 289–290. (10.1093/bioinformatics/btg412) 14734327

[44] R Development Core Team. 2012 R: a language and environment for statistical computing, 2.15.2 GUI 1.53 edn Vienna, Austria: R Foundation for Statistical Computing.

[45] Paradis E 2013 Molecular dating of phylogenies by likelihood methods: a comparison of models and a new information criterion. Mol. Phylogenet. Evol. 67, 436–444. (10.1016/j.ympev.2013.02.008) 23454091

[46] Kembel SW, Cowan PD, Helmus MR, Cornwell WK, Morlon H, Ackerly DD, Blomberg SP, Webb CO 2010 Picante: R tools for integrating phylogenies and ecology. Bioinformatics 26, 1463–1464. (10.1093/bioinformatics/btq166). 20395285

[47] Webb CO, Ackerly DD, McPeek MA, Donoghue MJ 2002 Phylogenies and community ecology. Annu. Rev. Ecol. Syst. 33, 475–505. (10.1146/annurev.ecolsys.33.010802.150448)

[48] Maddison WP, Maddison DT 2008 Mesquite: a modular system for evolutionary analysis, 2.5 edn. http://mesquiteproject.org

[49] Vorontsova MS, Rakotoarisoa SE 2014 Endemic non-bambusoid genera of grasses (Poaceae) in Madagascar: review of current knowledge. Malagasy Nat. 8, 14–34.

[50] Dransfield S 2003 Poaceae, Bambuseae, bamboos. In The natural history of Madagascar (eds Goodman SM, Benstead JP), pp. 467–471. Chicago, IL: University of Chicago Press.

[51] Callmander MW et al. 2011 The endemic and non-endemic vascular flora of Madagascar updated. Plant Ecol. Evol. 144, 121–125. (10.5091/plecevo.2011.513)

[52] Kier G, Kreft H, Lee TM, Jetz W, Ibisch PL, Nowicki C, Mutke J, Barthlott W 2009 A global assessment of endemism and species richness across island and mainland regions. Proc. Natl Acad. Sci. USA 106, 9322–9327. (10.1073/pnas.0810306106) 19470638 PMC2685248

[53] Buerki S, Devey DS, Callmander MW, Phillipson PB, Forest F 2013 Spatio-temporal history of the endemic genera of Madagascar. Bot. J. Linnean Soc. 171, 304–329. (10.1111/boj.12008)

[54] Crisp MD et al. 2009 Phylogenetic biome conservatism on a global scale. Nature 458, 754–756. (10.1038/nature07764) 19219025

[55] Graham CH, Fine PVA 2008 Phylogenetic beta diversity: linking ecological and evolutionary processes across space in time. Ecol. Lett. 11, 1265–1277. (10.1111/j.1461-0248.2008.01256.x) 19046358

[56] Holt BG et al. 2013 An update of Wallace's zoogeographic regions of the world. Science 339, 74–78. (10.1126/science.1228282) 23258408

[57] Hattab T, Albouy C, Ben Rais Lasram F, Le Loc'h F, Guilhaumon F, Leprieur F 2015 A biogeographical regionalization of coastal Mediterranean fishes. J. Biogeogr. 42, 1336–1348. (10.1111/jbi.12505)

[58] Nanjarisoa OP 2015 Inventaire taxonomique des Graminées dans la Nouvelle Aire Protégée du massif d'Itremo. Mémoire pour l'obtention du Diplôme d'Etude Approfondies (D.E.A) en Biologie et Ecologie Végétales. Madagascar, University d'Antananarivo.

[59] Morat P 1973 Les savanes du sud-ouest de Madagascar, vol. 68, 235 p. Paris, France: Mémoires Orstom.

[60] Lewis BA, Phillipson PB, Andrianarisata M, Rahajasoa G, Rakotomalaza PJ, Randriambololona M, McDonagh JF 1996 A study of the botanical structure, composition and diversity of the eastern slopes of the Reserve Naturelle Integrale d'Andringitra, Madagascar. Fieldiana 85, 24–75.

[61] Gautier L 1997 Inventaire floristique de la Reserve Speciale de Manongarivo (nord-ouest de Madagascar): Monocotyledonae. In *Documents EPB*, no. 5. Geneva, Switzerland: Institut universitaire détudes du développement.

[62] Vollesen K, Abdallah R, Coe M, Mboya E 1999 Checklist: vascular plants and pteridophytes of Mkomazi. In Mkomazi: the ecology, biodiversity and conservation of a Tanzanian savanna, pp. 81–116. London, UK: Royal Geographical Society.

[63] Vollesen K 1980 Annotated check-list of the vascular plants of the Selous Game Reserve, Tanzania. Opera Botanica 59, 1–117.

[64] Verdu M, Pausas JG 2007 Fire drives phylogenetic clustering in Mediterranean Basin woody plant communities. J. Ecol. 95, 1316–1323. (10.1111/j.1365-2745.2007.01300.x)

[65] Dinnage R 2009 Disturbance alters the phylogenetic composition and structure of plant communities in an old field system. PLoS ONE 4, e7071 (10.1371/journal.pone.0007071) 19763265 PMC2740862

[66] Helmus MR, Keller W, Paterson MJ, Yan ND, Cannon CH, Rusak JA 2010 Communities contain closely related species during ecosystem disturbance. Ecol. Lett. 13, 162–174. (10.1111/j.1461-0248.2009.01411.x). 20015255

[67] Goodman SM, Jungers WL 2014 Extinct Madagascar: picturing the island's past. Chicago, IL: University of Chicago Press.

[68] Crowley BE, Godfrey LR, Irwin MT 2011 A glance to the past: subfossils, stable isotopes, seed dispersal, and lemur species loss in Southern Madagascar. Am. J. Primatol. 73, 25–37. (10.1002/ajp.20817). 20205184

[69] Clarke SJ, Miller GH, Fogel ML, Chivas AR, Murray-Wallace CV 2006 The amino acid and stable isotope biogeochemistry of elephant bird (*Aepyornis*) eggshells from southern Madagascar. Quat. Sci. Rev. 25, 2343–2356. (10.1016/j.quascirev.2006.02.001)

[70] Burleigh R, Arnold EN 1986 Age and dietary differences of recently extinct Indian-Ocean Tortoises (*Geochelone* s lat) revealed by carbon isotope analysis. Proc. R. Soc. B 227, 137–144. (10.1098/rspb.1986.0014).

[71] Pedrono M, Griffiths OL, Clausen A, Smith LL, Griffiths CJ, Wilmé L, Burney DA 2013 Using a surviving lineage of Madagascar's vanished megafauna for ecological restoration. Biol. Conserv. 159, 501–506. (10.1016/j.biocon.2012.11.027)

[72] Godfrey LR, Jungers WL 2003 The extinct sloth lemurs of Madagascar. Evol. Anthropol. 12, 252–263.

