# Supplementary material for: Madagascar's grasses and grasslands: anthropogenic or natural?
Source: Proc Biol Sci. 2016 Jan 27;283(1823):20152262. doi: 10.1098/rspb.2015.2262 (PMC4795014; doi:10.1098/rspb.2015.2262)
Supplement: ESM Figures and Tables [file rspb20152262supp1.pdf]

Supporting information

Figure S1. The subfamily composition of the grass flora composition of East Africa (892 species) closely mirrors that of the Western Indian Ocean islands (611 species) and Madagascar only (541 species). Panicoideae are the most speciose. The only substantial difference is in the Bambusoideae, which are six times more common in Madagascar than in East Africa.

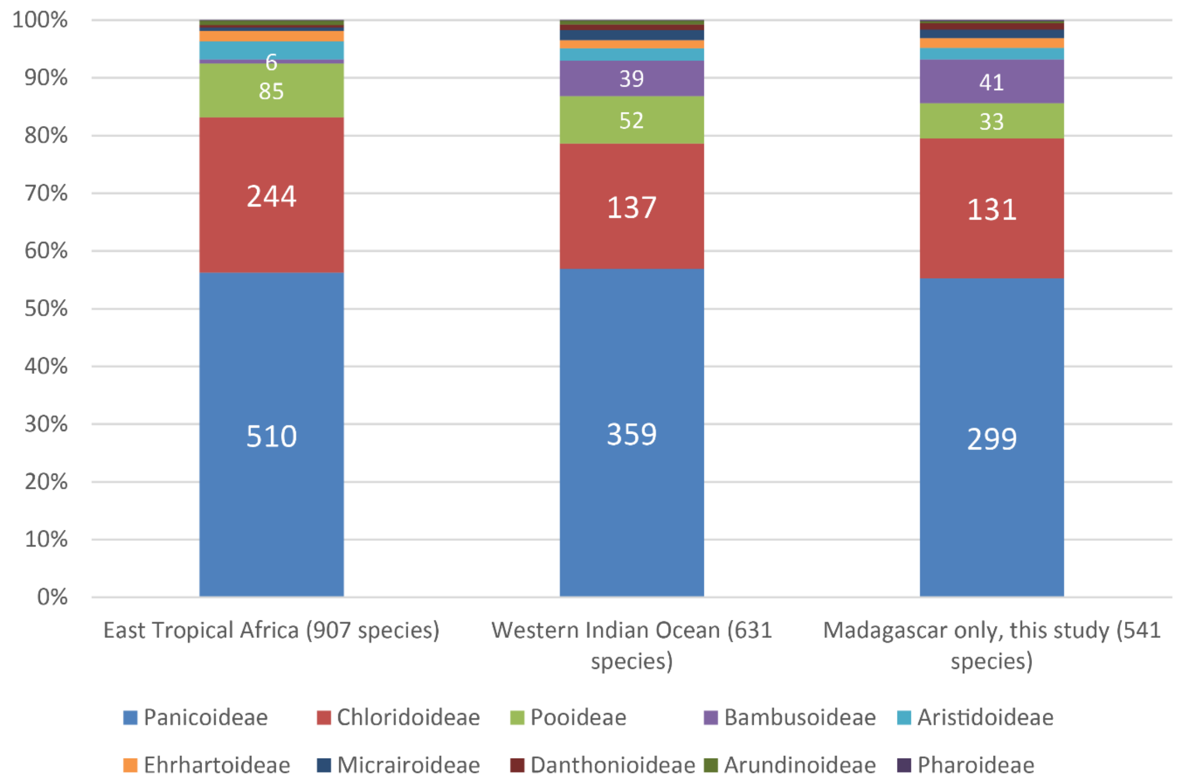

S1 Poaceae species composition sampling method

The sampling method was designed to capture the diversity of Poaceae and associated vegetation for a uniform vegetation type at each site. The centre plot 1 m in diameter was delineated using a plastic circle placed near the centre of a vegetation type minimum 60 m wide. Vegetation within the circle or overlapping the boundaries was examined carefully and a list of all Poaceae species was made. Care was taken to examine all grasses in the vegetative state, distinguish between these, and find nearby fertile individuals when possible to aid identification. Four transects 30 m each were placed in a radial pattern from the centre plot. Randomly generated numbers between 1 and 360 were used as angles from North to place the transects (Fig. S1). The circle was placed at 5m intervals along each transect and all Poaceae species were listed within the circle, or overlapping with the boundaries. When a transect was judged to cross into a different vegetation type it was terminated before the transition to ensure a single site represents a single community. When a vegetation type was linear, such as a stream bed or a cliff face, two transects of 30m each were placed in either direction from the centre plot. Vegetation types smaller than 60 m wide or 60 m long if linear were not included in this study. Local residents and protected area employees were interviewed about the history of the area focusing particularly on the history of disturbance, grazing, and burning. A herbarium voucher and silica gel collection was made of every grass species from the site including those in the vegetative state and those doubtfully distinct from other species. A list of associated species was made, and metadata was recorded including photographs, GPS data, elevation, geomorphology, bedrock, rockiness, proportion bedrock, slope, aspect, drainage, soil colour, soil depth, soil texture, soil organic content, general character of the vegetation, fire, and percentage grass cover.

Figure S2. Each study site was placed in a uniform vegetation type. All Poaceae species within circular plots at the centre of the site and along four transects were recorded.

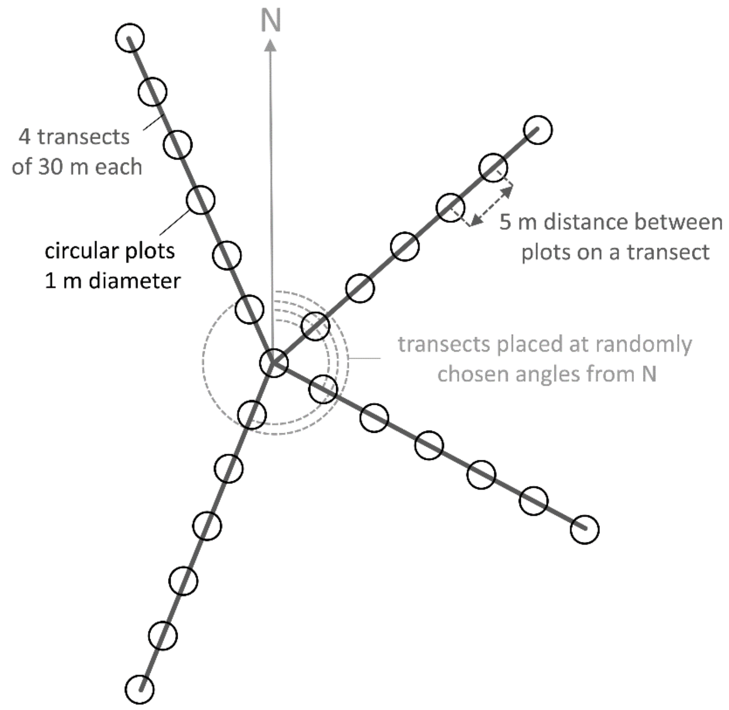

Table S1. Areas of the world used in Figure 2. The numbers indicate TDWG level 2 regions and amendments to this system.

Island regions
MDG Madagascar island only
21 Macaronesia
28 Middle Atlantic Ocean
29 Western Indian Ocean (including Madagascar)
38-2 Part of Eastern Asia: Japan
43-1 Part of Papuasia: Bismarck Archipelago, New Guinea
51 New Zealand
50 Australia
61 South-Central Pacific
60,43-2 Southwestern Pacific and part of Papuasia (Solomon Is.)
62 Northwestern Pacific
63 North-Central Pacific
81 Caribbean
90 Subantarctic islands

Continental regions
10 Northern Europe
11 Middle Europe
12-1 Part of Southwestern Europe: France
12-2 Part of Southwestern Europe: Portugal, Spain
13 Southeastern Europe
14-1 Part of Eastern Europe: Belarus, Baltic States, Central European Russia, North European Russia, Northwest European Russia
14-2 Part of Eastern Europe: Krym, East European Russia, South European Russia, Ukraine
20-1 Part of Northern Africa: Algeria, Libya, Morocco, Tunisia, Western Sahara
20-2,24-1 Part of Northern Africa (Egypt) and Northeast Tropical Africa (Chad, Sudan)
22 West Tropical Africa
23 West-Central Tropical Africa
24-2 Part of Northeast Tropical Africa: Djibouti, Eritrea, Ethiopia, Socotra, Somalia
25 East Tropical Africa
26-1,27-2 Part of South Tropical Africa (Angola, Malawi, Mozambique, Zambia, Zimbabwe) and part of Southern Africa (Botswana)
27-1 Part of Southern Africa: Cape Provinces, Caprivi Strip, Lesotho, Namibia, KwaZulu-Natal, Free State, Swaziland, Northern Provinces
31 Russian Far East
32,30-1 Central Asia and part of Siberia (Altay)
30-2 Part of Siberia: Buryatiya, Chita, Irkutsk, Tuva, Yakutiya
30-3 Part of Siberia: Krasnoyarsk, West Siberia
33 Caucasus
34,40-1 Western Asia and part of the Indian Subcontinent (Pakistan)
35 Arabian Peninsula
36-1 Part of China: Qinghai, Tibet, Xinjiang
36-2 Part of China: China South-Central, Hainan, China Southeast
36-2 Part of China:
36-3,37,38-1 Part of China (Inner Mongolia, Manchuria, China North-Central), Mongolia, and part of Eastern Asia (Korea)
40-2 Part of Indian Subcontinent: East Himalaya, Nepal, West Himalaya
40-3 Part of Indian Subcontinent: Assam, Bangladesh, India, Laccadive Is., Maldives, Sri Lanka
41 Indo-China
42 Malesia
70 Subarctic America
71 Western Canada
72 Eastern Canada
73 Northwestern USA
74 North-Cantral USA
75 Northeastern USA
76 Southwestern USA
77 South-Central USA
78 Southeastern USA
79 Mexico
80 Central America
82 Northern South America
83 Western South America
84-1 Part of Brazil: Brazil West-central, Brazil Northeast, Brasil Southeast
84-2 Part of Brazil: Brazil North
84-3,85-1 Part of Brazil (Brazil South) and part of Southern South America (Paraguay, Uruguay)
85 Southern South America

S2. Study sites and Poaceae species recorded at each site.

Site 2 "Pic Boby". Madagascar, Fianarantsoa, Andringitra National Park, directly below summit of Imarivolanitra (formally Pic Boby), between granite outcrops, 22.1949° S, 46.7585° E, Alt: 2650m, 12 December 2013. *Andropogon andringitrensis, Panicum* sp. nov.*, Pentameris andringitrensis.*

Site 3 "Poa stream". Madagascar, Fianarantsoa, Andringitra National Park, near the path building shelter ca 500m below summit of Imarivolanitra (formally Pic Boby), stream margin in Erica thicket with large boulders; shade under large granite boulder, 22.1949° S, 46.8899° E, Alt: 2470m, 12 December 2013. *Brachiaria dimorpha, Merxmuellera ambalavaoensis, Panicum cupressifolium, Poa perrieri.*

Site 4 "Panicum cupressifolium stand". Madagascar, Fianarantsoa, Andringitra National Park, below Imarivolanitra (formally pic Boby) along access path, level basin flanked by streams; Panicum cupressifolium grassland surrounded by Erica thicket, 22.1950° S, 46.8915° E, Alt: 2469m, 12 December 2013, *Brachiaria dimorpha, Merxmuellera ambalavaoensis, Panicum cupressifolium*.

Site 5 "Lecomtella stream". Madagascar, Fianarantsoa, Andringitra National Park, path above camp 3 to Imarivolanitra (formally pic Boby), streamline along mountainside; dense Erica thicket burned more than 10 years ago, 22.1864° S, 46.9007° E, Alt: 2220m, 13 December 2013, *Agrostis sp, Arundinella nepalensis, Brachiaria dimorpha, Brachypodium madagascariense, Lecomtella madagascariensis, Panicum andringitrense, Saccharum viguieri.*

Site 6 "Saccharum hillside". Madagascar, Fianarantsoa, Andringitra National Park, path above camp 3 to pic Boby, steep dry mountain slope; tall Erica thicket with much dead wood 2.5 m tall, 22.1927° S, 46.8998° E, Alt: 2261m, 13 December 2013, *Merxmuellera ambalavaoensis, Panicum andringitrensis, Saccharum viguieri*.

Site 7 "Sponge". Madagascar, Fianarantsoa, Andringitra National Park, basin east of camp 3, gentle colluvial inner basin traversed by streams; grassland surrounded by belts of Erica, 22.1922° S, 46.9056° E, Alt: 2054m, 13 December 2013, *Brachiaria dimorpha, Agrostis elliotii, Arundinella nepalensis, Panicum spergulifolium, Schizachyrium sanguineum.*

Site 8 "Erica thicket near sponge". Madagascar, Fianarantsoa, Andringitra National Park, plateau east of camp 3, gentle rounded hill; sparse 2m tall Erica scrub, 22.1911° S, 46.9059° E, Alt: 2070m, 13 December 2013, *Aristida tenuissima, Brachiaria dimorpha, Ctenium concinnum, Saccharum viguieri, Sporobolus centrifugus*.

Site 9 "Xerophyta hill". Madagascar, Fianarantsoa, Andringitra National Park, hills east of camp 3, rounded bedrock hill; grassy ericoid vegetation less than 1 m tall, 22.1890° S, 46.9073° E, Alt: 2096m, 13 December 2013, *Andropogon andringitrensis, Aristida tenuissima, Panicum spergulifolium.*

Site 10 "Erica thicket". Madagascar, Fianarantsoa, Andringitra National Park, at camp 2, gentle slope; 2 m tall Erica thicket, 22.1496° S, 46.9004° E, Alt: 2004m, 14 December 2013, *Panicum perrieri, Agrostis elliotii, Andropogon andringitrensis, Aristida tenuissima, Brachiaria dimorpha, Ctenium concinnum, Elionurus tristis, Loudetia simplex, Panicum mitopus*, indet. 1, indet. 2,

Site 11 "Three year old Erica". Madagascar, Fianarantsoa, Andringitra National Park, at camp 2, gentle slope; Erica thicket, young Erica 0.5-1 m tall, 22.1513° S, 46.9004° E, Alt: 1985m, 14 December 2013, *Agrostis elliotii, Aristida tenuissima, Brachiaria dimorpha, Elionurus tristis, Festuca camusiana, Sporobolus centrifugus*, indet. 3, indet. 4.

Site 12 "One year old Erica". Madagascar, Fianarantsoa, Andringitra National Park, at camp 2, gentle hill above stream, burnt, first year herbaceoaus regrowth, 22.1525° S, 46.8975° E, Alt: 1985m, 14

December 2013, *Agrostis elliotii, Aristida tenuissima, Brachiaria epacridifolia, Ctenium concinnum, Elionurus tristis, Panicum perrieri, Sporobolus centrifugus.*

Site 13 "Afromontane forest". Madagascar, Fianarantsoa, Andringitra National Park, steep slopes next to base of Riambavy waterfall between camps 1 and 2, low afromontane evergreen forest; steep granitic slope, 22.1418° S, 46.8954° E, Alt: 1822m, 14 December 2013, *Brachypodium madagascariense, Oplismenus hirtellus*, indet. 5.

Site 14 "Namaza stream bank". Madagascar, Toliara, Isalo National Park, Namaza River bank above Namaza camp, rocky high energy stream; Pandanus thicket, 22.5389° S, 45.3785° E, Alt: 780m, 16 December 2013, *Andropogon eucomus, Aristida similis, Digitaria longiflora, Ischaemum koleostachys, Panicum brevifolium, Panicum hymeniochilum, Phragmites mauritianus, Setaria sphacelata, Trichanthecium parvifolium, Trichopteryx dregeana.*

Site 15 "Isalo top rock". Madagascar, Toliara, Isalo National Park, path to the Blue and Black Pools, top of escarpment above Namaza valley, eroded sandstone outcrops on top of sandstone plateau; grassland with occasional shrubs partially burnt in 2010, 22.5377° S, 45.3748° E, Alt: 897m, 17 December 2013, *Craspedorhachis africana, Loudetia simplex, Styppeiochloa hitchcockii, Trachypogon spicatus, Tristachya isaloensis.*

Site 16 "Isalo tapia". Madagascar, Toliara, Isalo National Park, path to the Blue and Black Pools, plateau above town spring, tapia savanna on sandstone plateau, 3 years after burning, 22.5363° S, 45.3732° E, Alt: 898m, 17 December 2013, *Andropogon ibityensis, Craspedorhachis africana, Heteropogon contortus, Loudetia simplex, Styppeiochloa hitchcockii, Trachypogon spicatus, Tristachya isaloensis.*

Site 17 "Bamboo forest". Madagascar, Toliara, Isalo National Park, steep slope above Namaza stream, steep slope between cliffs and river; humid forest and bamboo thicket, 22.5390° S, 45.3780° E, Alt: 784m, 18 December 2013, *Isachne mauritiana, Oplismenus compositus, Valiha sp.*

Site 18 "Sartidia ledge". Madagascar, Toliara, Isalo National Park, 1.7 km from Namaza camp on the trail to the Blue and Black Pools, ledge above Waterfall of the Nymphs on route to town spring, vertical sandstone wall; occasional grass in rock pockets and ledges, 22.5379° S, 45.3772° E, Alt: 885m, 18 December 2013, *Sartidia isaloensis, Styppeiochloa hitchcockii, Tristachya isaloensis.*

Site 19 "No grass forest". Madagascar, Toliara, Isalo National Park, long path just before Namaza camp from the car park, seasonally dry scrub forest; gentle slope above stream, 22.5398° S, 45.3813° E, Alt: 790m, 18 December 2013, *Hyparrhenia newtonii, Oplismenus compositus, Urochloa maxima.*

Site 20 "Ambalavao slope". Madagascar, Fianarantsoa, Matsiatra Ambony, Sambiraty, 2 km S from village Ambaiboho on RN7, from Ambalavao to Ihosy, Loudetia simplex grassland ca 1 m tall on rocky slope, 21.8638° S, 46.8107° E, Alt: 1075m, 19 April 2014, *Aristida similis, Chrysopogon serrulatus, Cymbopogon caesius, Eragrostis lateritica, Heteropogon contortus, Hyparrhenia rufa, Loudetia simplex, Paspalum scrobiculatum.*

Site 21 "Horombe plateau". Madagascar, Fianarantsoa, Ihorombe, middle of the Horombe plateau ca 300 m away from the RN7, short sparse Trachypogon grassland up to 1 m, burned ca 1 year ago, 22.4527° S, 45.8428° E, Alt: 1016m, 19 April 2014, *Heteropogon contortus, Hyparrhenia rufa, Loudetia simplex, Melinis repens, Trachypogon spicatus.*

Site 22 "Trachypogon grassland outside Isalo". Madagascar, Fianarantsoa, Ihorombe, ca 5 km from Ranohira towards Llakaka, Trachypogon open grassland 1.5 m tall, flat plane between sandstone outcrops, 22.6267° S, 45.3495° E, Alt: 819m, 20 April 2014, *Chrysopogon serrulatus, Craspedorhachis*

africana, Loudetia filifolia, Loudetia simplex, Panicum cinctum, Schizachyrium sanguineum, Trachypogon spicatus, Tristachya isalensis, Urelytrum agropyroides.

Site 23 "Tsimanampetsotsa dry forest". Madagascar, Toliara, Lac Tsimanampetsotsa National Park; near the viewpoint closest to the camping site, dry forest 2m tall dominated by Cedrellopsis, on eroded limestone karst, 24.0884° S, 43.7566° E, Alt: 17m, 24 April 2014, *Acroceras calcicola, Brachiaria humbertiana, Dactyloctenium capitatum, Enneapogon cenchroides, Eragrostis capuronii, Lepturus anadabolavensis.*

Site 24 "Tsimanampetsotsa escarpment forest". Madagascar, Toliara, Lac Tsimanampetsotsa National Park; Soarano, 13 km from Tsimaso viewpoint, dry forest 2m tall with Commiphora and Cedrelopsis; eroded limestone escarpment above lake, 24.1856° S, 43.7722° E, Alt: 74m, 24 April 2014, *Aristida adscensionis, Eragrostis capuronii, Lepturus anadabolavensis*.

Site 25 "Tsimanampetsotsa salt plane". Madagascar, Toliara, Lac Tsimanampetsotsa National Park; Soarano, N end of lake Tsimanampetsotsa, salt flat grazed by zebu daily, 24.1893° S, 43.7706° E, Alt: 9m, 24 April 2014, *Sporobolus virginicus, Paspalum vaginatum*.

Site 26 "Euphorbia stenophylla pasture". Madagascar, Toliara, 25 km N of Itampolo on the road from Tsimanampesotsa, Euphorbia stenophylla heavily grazed bushland; only P. voeltzkowii and Dactyloctenium survive the heavy grazing, 24.2226° S, 43.6857° E, Alt: 8m, 25 April 2014, *Dactyloctenium capitatum, Eragrostis minor, Lepturus anadabolavensis, Panicum voeltzkowii, Setaria humbertiana.*

Site 27 "Decaryella". Madagascar, Belamoty; route from delta of the Linta to Ampanihy, ca 5 km after turn off from the coastal road inland, spiny forest with Allaudia comosa and A.procera to 10 m, with unusually green understorey; grazed by goats and zebu daily, 25.0492° S, 44.3908° E, Alt: 160m, 25 April 2014, *Aristida adscensionis, Aristida barbicollis, Brachiaria humbertiana, Dactyloctenium capitatum, Decaryella madagascariensis, Eragrostis capuronii, Lepturus humbertianus, Panicum voeltzkowii, Urochloa maxima.*

Site 28 "Chrysopogon humbertianus". Madagascar, Toliara, ca 2 km from Tsihombe towards Cape Ste Marie, escarpment above the sea; dwarf shrubland 30 cm tall dominated by Megistostegium nodulosum, with high winds; occasionally visited by zebu who eat Aristida but not Chrysopogon, 25.5843° S, 45.1398° E, Alt: 187m, 27 April 2014, *Aristida adscensionis, Chrysopogon humbertianus, Setaria humbertiana, Sporobolus perrieri*

Site 29 "Eragrostis boinensis pasture". Madagascar, Toliara, Ex-Bevazoa, ca 3 km from Cape Ste Marie towards the ANGAP office, village Bevzoa moved from here in the 1960s, coastal forest pasture with Opuntia dominated by 2 m tall Megostotegium microphyllum, grazed daily by zeby and goats, 25.5840° S, 45.1421° E, Alt: 186m, 27 April 2014, *Digitaria didactyla, Eragrostis humbertii, Panicum voeltzkowii, Setaria humbertiana.*

Site 30 "Sclerodactylon beach". Madagascar, Toliara, Vavaony beach, immediately W of the Ste Marie grotte, sandy beach dominated by Sclerodactylon and Sporobolus virginicus; occasionally grazed by zebu, 25.6050° S, 45.1628° E, Alt: 11m, 28 April 2014, *Dactyloctenium capitatum, Panicum voeltzkowii, Sclerodactylon macrostachyum, Sporobolus perrieri, Sporobolus virginicus.*

Site 31 "Chloris humbertiana spiny forest". Madagascar, Toliara, Amiboroda, 2.5 km from Cape Ste Marie ANGAP office on the road to Soamanitra and Lavanono, spiny forest 3m tall dominated by Operculicarya and Euphorbia stenoclada; grazed by zebu and goat daily, 25.5189° S, 45.1340° E, Alt: 154m, 28 April 2014, *Chloris humbertiana, Dactyloctenium capitatum, Eragrostis capuronii, Eragrostis minor, Setaria humbertiana.*

Site 32 "Ste Marie fallow field". Madagascar, Toliara, Ambalanosy, 3 km from Cape Ste Marie ANGAP office on the road to Soamanitra and Lavanono, open grazed field fallow for 2 years out of 5 year rest period; maize, beans, sweet potato normally cultivated, 25.5061° S, 45.1158° E, Alt: 160m, 28 April 2014, *Brachiaria distachya, Cenchrus ciliaris, Cynodon radiatus, Dactyloctenium capitatum, Digitaria ciliaris, Digitaria didactyla, Eragrostis capuronii, Eragrostis humbertii, Panicum voeltzkowii.*

Site 33 "Decaryella 2". Madagascar, Toliara, Ambaliandro, 17 km before Tsihombe on the road from Cape Ste Marie to Tsihombe, mature spiny forest 3 m on deep red sand, regularly grazed by zebu, Euphorbia stenophylla harvested for charcoal, 25.3529° S, 45.4369° E, Alt: 128m, 29 April 2014, *Acroceras calcicola, Aristida adscensionis, Brachiaria humbertiana, Chloris humbertiana, Decaryella madagascariensis, Eragrostis capuronii, Eragrostis tenella, Lepturus humbertianus, Panicum voeltzkowii.*

Site 34 "Panicum malacotrichum forest fragment". Madagascar, Majunga, Sofia, Anjojahely, 69 km before Ambanja on RN6, remnant dry forest patch with Xylolaena; cut 2-3 years ago; grazed by zebu, 14.0861° S, 48.1094° E, Alt: 225m, 9 May 2014, *Brachiaria bemarivensis, Olyra latifolia, Oplismenus burmannii, Panicum malacotrichum.*

Site 35 "Panicum malacotrichum forest gap". Madagascar, Antsiranana, Diana, Reserve Speciale Manongarivo, Bekolosy forest, 1.5 km N of Ambalifary, natural gap in humid mid-elevation forest dominated by Calophyllum over 15 m; no grazing, 14.0509° S, 48.2940° E, Alt: 788m, 12 May 2014, *Isachne mauritiana, Panicum malacotrichum, Sirochloa parvifolia.*

Site 36 "Nastus aristatus forest". Madagascar, Antsiranana, Diana, Reserve Speciale Manongarivo, Bekolosy forest 2 km N of Ambalifary, open montane forest, never grazed; steep forested slope, 14.0478° S, 48.2958° E, Alt: 1055m, 12 May 2014, *Nastus aristatus, Sirochloa parvifolia.*

Site 37 "Panicum vohitrense granite wall". Madagascar, Antsiranana, Diana, Reserve Speciale Manongarivo, Bekolosy forest 2 km N of Ambalifary, mossy granite surface under mature forest canopy, no grazing, 14.0473° S, 48.2960° E, Alt: 1075m, 12 May 2014, *Panicum vohitrense, Sirochloa parvifolia.*

Site 38 "Poecilostachys and Dimeria riverside". Madagascar, Antsiranana, Diana, Reserve Speciale Manongarivo, Bekolosy river 2.3 km N of Ambalifary, granite slope above montane river; herbaceous mat on moss, visited by researchers only, 14.0455° S, 48.2964° E, Alt: 1135m, 12 May 2014, *Brachiaria bemarivensis, Dimeria manongarivensis, Oplismenus* sp. nov.

Site 39 "Manongarivo Aristida pasture". Madagascar, Antsiranana, Ankijanabe, between Reserve Speciale Manongarivo and RN6, 1km E of Antsambalahy village, 500 m S of river Antsambalahy, open grassland pasture 50 cm tall with Aristida similis and Hyparrhenia rufa, frequently grazed, 14.0513° S, 48.2314° E, Alt: 148m, 13 May 2014, *Aristida similis, Eragrostis chapelieri, Hyparrhenia rufa, Panicum brevifolium, Schizachyrium sanguineum.*

Site 40 "open Mascarenhasia forest". Madagascar, Antsiranana, Diana, between Reserve Speciale Manongarivo and RN6, 300 m S of river Antsambalahy, open woodland dominated by 3m tall Mascarenhasia, grazed by zebu and goats, 14.0499° S, 48.2277° E, Alt: 110m, 13 May 2014, *Aristida similis, Brachiaria umbellata, Cenchrus polystachion, Hyparrhenia rufa, Panicum brevifolium, Paspalum scrobiculatum, Sacciolepis curvata , Schizachyrium sanguineum, Viguierella madagascariensis.*

Site 41 "Valiha slope". Madagascar, Antsiranana, Diana, RN6 roadside, 32 km S of Ambanja, just before village Ambodibonga travelling from Ambanja, bamboo thicket ca 4 m, with no sign of grazing,

13.8563° S, 48.2667° E, Alt: 81m, 14 May 2014, *Hyparrhenia rufa, Oplismenus burmannii, Panicum brevifolium, Paspalum conjugatum, Valiha diffusa.*

Site 42 "Ankaramy Be pasture". Madagascar, Mahajanga, Sofia, 1 km S of Amkaramy Be on RN6, open Aristida similis grassland ca 40 cm, likely heavily grazed, 13.9897° S, 48.1749° E, Alt: 156m, 14 May 2014, *Aristida similis, Cenchrus polystachion, Eragrostis chapelieri, Heteropogon contortus, Hyparrhenia rufa, Panicum brevifolium, Schizachyrium sanguineum.*

Site 43 "Ambatofinandrahana pine hillside". Madagascar, Fianarantsoa, Ambatofinandrahana, Andriantantely, Magrama, road to Magrama marble factory ca 1 km S of Ambatofinandrahana, undulating terrain with limestone outcrops; grazed grassland in an open Pine stand, 20.5763° S, 46.8121° E, Alt: 1360m, 18 May 2014, *Andropogon trichozygus, Aristida similis, Chrysopogon serrulatus, Cymbopogon caesius, Digitaria longiflora, Digitaria pseudodiagonalis, Elionurus tristis, Heteropogon contortus, Hyparrhenia newtonii, Hyparrhenia rufa, Loudetia simplex, Paspalum scrobiculatum, Schizachyrium brevifolium, Schizachyrium sanguineum, Setaria sphacelata, Sporobolus centrifugus, Trachypogon spicatus, Urelytrum agropyroides.*

Site 44 "Ihazafotsy village pasture". Madagascar, Fianarantsoa, Ambatofinandrahana, 200 m above Ihazafotsy village, just outside NAP Itremo, open valley between limestone hills; heavily grazed open pasture ca 15 cm tall, 20.5813° S, 46.6115° E, Alt: 1297m, 19 May 2014, *Andropogon trichozygus, Aristida similis, Digitaria longiflora, Loudetia simplex, Schizachyrium sanguineum, Sporobolus centrifugus, Urelytrum agropyroides.*

Site 45 "Saccharum perrieri stream". Madagascar, Fianarantsoa, Ambatofinandrahana, Itremo NAP, Anovitrinimpirahavavy stream, Antsirakambiaty, stream below the end of car track to Antsirakambiaty, dense open scrub 2 m above stream, dominated by 2m tall Hyparrhenia schimperi; no grazing, 20.5958° S, 46.5630° E, Alt: 1516m, 19 May 2014, *Arundinaria sp. nov., Arundinella nepalensis, Brachypodium madagascariense, Calamagrostis emirnenis, Digitaria atrofusca, Festuca camusiana, Heteropogon melanocarpus, Hyparrhenia rufa, Hyparrhenia schimperi, Ischaemum polystachyum, Panicum hymeniochilum, Saccharum perrieri, Urelytrum agropyroides.*

Site 46 "High Plateau forest with Arundinaria". Madagascar, Fianarantsoa, Ambatofinandrahana, Itremo NAP, Antsirakambiaty forest, narrow rocky valley bottom; mature high plateau gallery forest, habitat of Mantella cavanii, no grazing, 20.5969° S, 46.5619° E, Alt: 1550m, 19 May 2014, *Arundinaria* sp. nov., *Hickelia madagascariensis, Isachne mauritiana, Oplismenus compositus, Oplismenus flavicomus, Panicum mitopus, Pseudobromus breviligulatus.*

Site 47 "Degraded tapia forest". Madagascar, Fianarantsoa, Ambatofinandrahana, Itremo NAP, Ambatoasira tapia forest, top of limestone hill; open degraded tapia forest ca 5 m with Leptolaena, Sarcolaena, and silk moth cacoons; grazed weekly; tapia speading due to reduced fire regime, 20.6121° S, 46.5739° E, Alt: 1666m, 20 May 2014, *Agrostis elliotii, Andropogon itremoensis, Aristida similis, Cyrtococcum deltoideum, Digitaria longiflora, Digitaria pseudodiagonalis, Eragrostis lateritica, Festuca camusiana, Hyparrhenia newtonii, Hyparrhenia rufa, Loudetia simplex, Panicum ibityense, Panicum perrieri, Paspalum scrobiculatum, Schizachyrium sanguineum, Setaria sphacelata, Sporobolus centrifugus, Sporobolus piliferus, Trachypogon spicatus, Urelytrum agropyroides.*

Site 48 "Ianasana campsite mountain stream". Madagascar, Fianarantsoa, Ambatofinandrahana, Itremo NAP, 300 m below the Ianasana campsite, stream at the valley bottom of steep limestone outcrops; low vegetation with Pinus, 20.5795° S, 46.5882° E, Alt: 1463m, 20 May 2014, *Aristida similis, Arundinella nepalensis, Ctenium concinnum, Ischaemum polystachyum, Loudetia simplex, Panicum subhystrix, Schizachyrium sanguineum, Trachypogon spicatus.*

Site 49. “Styppeiochloa rocks above Ianasana Madagascar”. Fianarantsoa, Ambatofinandrahana, Itremo NAP, Ianasana campsite, open low herbaceous vegetation on quartz with Pachypodium and Euphorbia calcicola, frequently grazed, 20.5788° S, 46.5894° E, Alt: 1486m, 20 May 2014, *Loudetia simplex, Panicum subhystrix, Schizachyrium sanguineum, Sporobolus centrifugus, Styppeiochloa hitchcockii, Trachypogon spicatus.*

Site 50 “Hanang Protea hillside”. Tanzania, Arusha, Hanang Mountain, Udamaschek, Jerdom village valley, gentle mountainside slope; bushed grassland with Protea and Kotschya; grazing by hares only; accidental burn 3 years ago, 4.4596° S, 35.3904° E, Alt: 2772m, 13 June 2014, *Agrostis producta, Andropogon chrysostachyus, Eragrostis schweinfurthii, Exotheca abyssinica, Festuca abyssinica, Helictotrichon elongatum, Setaria sphacelata.*

Site 51 “Hanang Protea ridge”. Tanzania, Arusha, Hanang Mountain, Udamaschek, Gabado village territory, gentle mountainside slope; bushed grassland; no grazing; burned 1 year ago, 4.4524° S, 35.3875° E, Alt: 3079m, 13 June 2014, *Agrostis producta, Andropogon amethystinus, Bromus leptoclados, Eragrostis schweinfurthii, Exotheca abyssinica, Festuca abyssinica, Festuca obturbans, Helictotrichon elongatum, Pentameris borussica, Poa leptoclada, Vulpia bromoides.*

Site 52 “Hanang Anthoxanthum nivale”. Tanzania, Arusha, Hanang Mountain, 2.2km before summit, mountain ridge; dense Anthospermum thicket 1m; no grazing; fire 10 years ago, 4.4468° S, 35.3892° E, Alt: 3199m, 14 June 2014, *Agrostis producta, Andropogon amethystinus, Anthoxanthum nivale, Exotheca abyssinica, Festuca abyssinica, Festuca obturbans, Poa leptoclada.*

Site 53 “Hanang Ehrharta forest”. Tanzania, Arusha, Hanang Mountain, Dahari forest, 4km S of Hanang peak, gentle montane slope; dry forest with Juniperus procera and Olinia rochetiana; firewood arvesting with machete forbidden; no grazing; no fire, 4.4778° S, 35.3964° E, Alt: 2143m, 14 June 2014, *Bothriochloa* sp.*, Brachypodium flexum, Bromus leptoclados, Ehrharta erecta* var. *abyssinica, Hyparrhenia cymbaria, Oplismenus compositus, Panicum wiehei, Setaria sphacelata.*

Site 54 “Hanang pasture”. Tanzania, Arusha, Hanang Mountain, Jerdom valley, Udamaschek village, pasture 1m tall dominated by Themeda, never cultivated, forest cleared many years ago; never burned, 4.4975° S, 35.3959° E, Alt: 1979m, 15 June 2014, *Aristida adoensis, Bothriochloa insculpta, Brachiaria brizantha, Brachiaria semiundulata, Digitaria abyssinica, Digitaria velutina, Eragrostis racemosa, Hyparrhenia anamesa, Hyparrhenia collina, Setaria incrassata, Setaria sphacelata, Sporobolus pyramidalis, Themeda triandra.*

Site 55 “Uzondo grassland with Aristida humidicola”. Tanzania, Rukwa, Mpanda, Uzondo Plateau S of Uvinza, road from Mpanda, flat plateau; open grassland; no grazing, 5.4839° S, 30.5300° E, Alt: 1665m, 21 June 2014, *Anadelphia scyphofera, Andropogon africanus, Andropogon canaliculatus, Aristida humidicola, Brachiaria brizantha, Diheteropogon amplectens var. catangensis, Eragrostis canescens, Eragrostis dentifera, Exotheca abyssinica, Hyparrhenia bracteata, Loudetia lanata, Microchloa kunthii, Setaria sphacelata, Trichanthecium brazzavillense, Trichanthecium tenellum.*

Site 56 “Uzondo campsite”. Tanzania, Rukwa, Mpanda, Uzondo Plateau S of Uvinza, road from Mpanda, open plateau; Loudetia grassland 2m tall, cow grazing, 5.4842° S, 32.5533° E, Alt: 1670m, 21 June 2014, *Andropogon africanus, Andropogon canaliculatus, Diheteropogon amplectens* var. *catangensis, Entolasia imbricata, Eragrostis canescens, Exotheca abyssinica, Loudetia lanata, Monocymbium ceresiiforme, Panicum flacciflorum, Setaria sphacelata, Trichanthecium brazzavillense, Trichanthecium tenellum.*

Site 61 “Suye hill top”. Tanzania, Arusha, top of Suye Hill, within Arusha city, degraded Afromontane forest used for regular fodder harvest, dominated by Albizzia gummifera, Croton megalocarpus,

Turraea robusta, and Clausena anisata, 3.3783° S, 36.7123° E, Alt: 1544m, 16 December 2014, *Cynodon nlemfuensis* var. *robustius, Digitaria velutina, Eleusine indica, Setaria megaphylla, Sporobolus agrostoides, Urochloa brachyura.*

Site 62 "Suye forest". Tanzania, Arusha, forest just above Karama lodge, Suye Hill, mature afromontane forest with invasive species, open understorey, dominated by Senna spectabilis, Grevillea robusta, and Croton megalocarpus, 3.3779° S, 36.7138° E, Alt: 1409m, 17 December 2014, *Oplismenus compositus, Panicum trichocladum, Setaria megaphylla, Sporobolus agrostoides.*

Site 63 "Suye fallow field". Tanzania, Arusha, Suye hill fallow field just before the entrance to Karama lodge, fallow filed 1-2 years after maize cultivation, 3.3811° S, 36.7153° E, Alt: 1405m, 17 December 2014, *Digitaria abyssinica, Digitaria velutina, Melinis repens, Panicum trichocladum, Setaria homonyma, Setaria verticillata, Sorghum arundinaceum.*

Site 64 "Monduli forest". Tanzania, Arusha, Escona forest, Mount Monduli, mature Afromontane forest dominatd by Euphorbia candelabra and Vangueria infausta, disturbed and regularly grazed, 3.2858° S, 36.4159° E, Alt: 1613m, 18 December 2014, *Digitaria velutina, Oplismenus compositus, Panicum deustum, Panicum infestum, Setaria megaphylla, Sporobolus agrostoides.*

Site 65 "Monduli Masai pasture". Tanzania, Arusha, Monduli Juu pasture, overgrazed short bushland on alluvial plane, 3.2580° S, 36.3943° E, Alt: 1600m, 18 December 2014, *Chloris pycnothrix, Cynodon dactylon, Digitaria abyssinica, Digitaria milanjiana, Digitaria velutina, Eragrostis tenuifolia, Themeda triandra.*

Site 66 "Monduli police ground". Tanzania, Arusha, Monduli town police ground football pitch, manually maintained open sports ground bordered by Jacaranda trees, usually not grazed, 3.2961° S, 36.4466° E, Alt: 1563m, 19 December 2014, *Bothriochloa insculpta, Chloris pycnothrix, Cynodon nlemfuensis* var. *robustius, Digitaria abyssinica, Digitaria velutina, Eleusine multiflora, Eragrostis tenuifolia, Urochloa brachyura,* indet. 6.

Site 67 "Mampikony white sand quarry". Madagascar, Mahajanga, RN6 from Mampikony to Ambanja; near a village, open bottom of sand quarry with pioneer vegetation dominated by Phialiphora; sand last harvested within the last 12 months; never burned, 15.4669° S, 47.5953° E, Alt: 117m, 21 February 2015, *Aristida similis, Brachiaria bemarivensis, Digitaria ciliaris, Eragrostis boinensis, Eragrostis gangetica,* indet. 7.

Site 68 "Mampikony white sand forest". Madagascar, Mahajanga, RN6 from Mampikony to Ambanja; directly above quarry site 67; near a village, western dry forest dominated by Croton melanostichtum and Sapium; wood regularly harvested; fire ca 1 year ago; recent charcoal visible, 15.4669° S, 47.5953° E, Alt: 117m, 21 February 2015, *Aristida similis, Brachiaria bemarivensis, Eragrostis boinensis, Neostapfiella perrieri.*

Site 69 "Nosy Be forest clearing". Madagascar, Antsiranana, Nosy-Be, northen Nosy Be adjacent to the ring road, clearing in sublittoral forest, between Cocos nucifera plantations, cleared for at least 5 years; burned ca 3 years ago, 13.2625° S, 48.2715° E, Alt: 122m, 22 February 2015, *Brachiaria umbellata, Digitaria longiflora, Lepturus radicans, Paspalum scrobiculatum.*

Site 70 "Lokobe Nastus forest". Madagascar, Antsiranana, Nosy-Be, Lokobe National Park, track above Circuit Ramy on the path towards 430 m peak, primary coastal wet evergreen forest; no disturbance; forestry low threat level, 13.4134° S, 48.3120° E, Alt: 187m, 23 February 2015, *Olyra latifolia, Sirochloa parvifolia.*

Site 71 “Lokobe forest below bamboo layer”. Madagascar, Antsiranana, Nosy Be, Lokobe National Park, track above Circuit Ramy on the path towards 430 m peak, below the bamboo, primary evergreen forest with Tambourissa and Canarium madagascariense, no disturbance; forestry low threat level, 13.4147° S, 48.3099° E, Alt: 82m, 23 February 2015, *Olyra latifolia*.

Site 72 “Ambatozavavy village games field”. Madagascar, Antsiranana, Nosy-Be, Ambatozavavy village games field; flat plane immediately behind coast, annually flooding village games field, forested prior to 1982, sand added and trees removed, grazing forbidden but regular, 13.3688° S, 48.3174° E, Alt: 1m, 24 February 2015, *Brachiaria hubbardii, Brachiaria umbellata, Cynodon dactylon, Echinochloa colona, Eleusine indica, Paspalum scrobiculatum, Sporobolus pyramidalis.*

Site 73 “Ambanja old cashew plantation”. Madagascar, Antsiranana, 15 km N of Ambanja on the RN6, secondary deciduous forest in an abandoned cashew nut plantation, dominated by Anacardium occidentale, last burned more than 10 years ago (Anacardium not burned), 13.5966° S, 48.5171° E, Alt: 31m, 25 February 2015, *Aristida similis, Brachiaria bemarivensis, Setaria madecassa.*

Site 74 “Ambilobe Eucalyptus plantation”. Madagascar, Antsiranana, N of Ambilobe on the RN6, Eucalyptus plantation with occasional Anacardium, frequently grazed, burned ca 3 years ago, 13.1514° S, 49.0747° E, Alt: 13m, 26 February 2015, *Alloteropsis semialata, Aristida similis, Brachiaria distachya, Digitaria longiflora, Eragrostis chapelieri, Heteropogon contortus, Paspalum scrobiculatum, Themeda quadrivalvis, Viguierella madagascariensis.*

Site 75 “Ankarana Campement des Anglais”. Madagascar, Antsiranana, Ankarana National Park, Encampment des Anglais, canyon between limestone tsingy, semi-deciduous primary forest on basalt, logging stopped since 1985, camp established in 1992, now visited by tourists and researchers only, 12.9086° S, 49.1100° E, Alt: 102m, 27 February 2015, *Humbertochloa bambusicuscula, Olyra latifolia.*

Site 76 “Ankarana Chasechloa forest”. Madagascar, Antsiranana, Ankarana National Park, path to Ambohimalaza viewpoint, 1km from the road leading to Encampment des Princes, tropophile forest, deciduous with partly open canopy ca 10m, with limited illegal logging and path clearing for tourists, 12.9688° S, 49.1195° E, Alt: 74m, 28 February 2015, *Chasechloa humbertiana*.

Site 77 “Ankarana savanna”. Madagascar, Antsiranana, Ankarana National Park, path to Ambohimalaza viewpoint, 2km from the road leading to Encampment des Princes, forest gap savanna with forest trees, Acacia and Flacourtia, commonly grazed, burned annually, 12.9741° S, 49.1144° E, Alt: 95m, 28 February 2015, *Heteropogon contortus, Hyparrhenia rufa.*

Site 78 “Diego Themeda hills”. Madagascar, Antsiranana, outside Montagne d'Ambre Parcelle 1; commune Antsahampano, fokontany Antongombato, locality Antamotamo, open grassland pasture burned every 1-2 years, 12.3796° S, 49.2054° E, Alt: 61m, 3 March 2015, *Dichanthium aristatum, Themeda quadrivalvis, Urochloa reptans.*

Fig. S3. Examples of undisturbed and highly disturbed sites studied in Madagascar. A site 4; B site 12; C site 46; D site 44; E site 38; F site 39; G site 24; H site 26. A, C, E, G are natural vegetation sites with disturbance scores 0. B, F suffer some disturbance with a score of 0.5. D, H are daily use pastures with a disturbance score of 1. A, B are in the alpine ecoregion; C, D are in the plateau ecoregion; E, F are in the Sambirano ecoregion; and G, H are in the south ecoregion. For full details of the sites see S2.

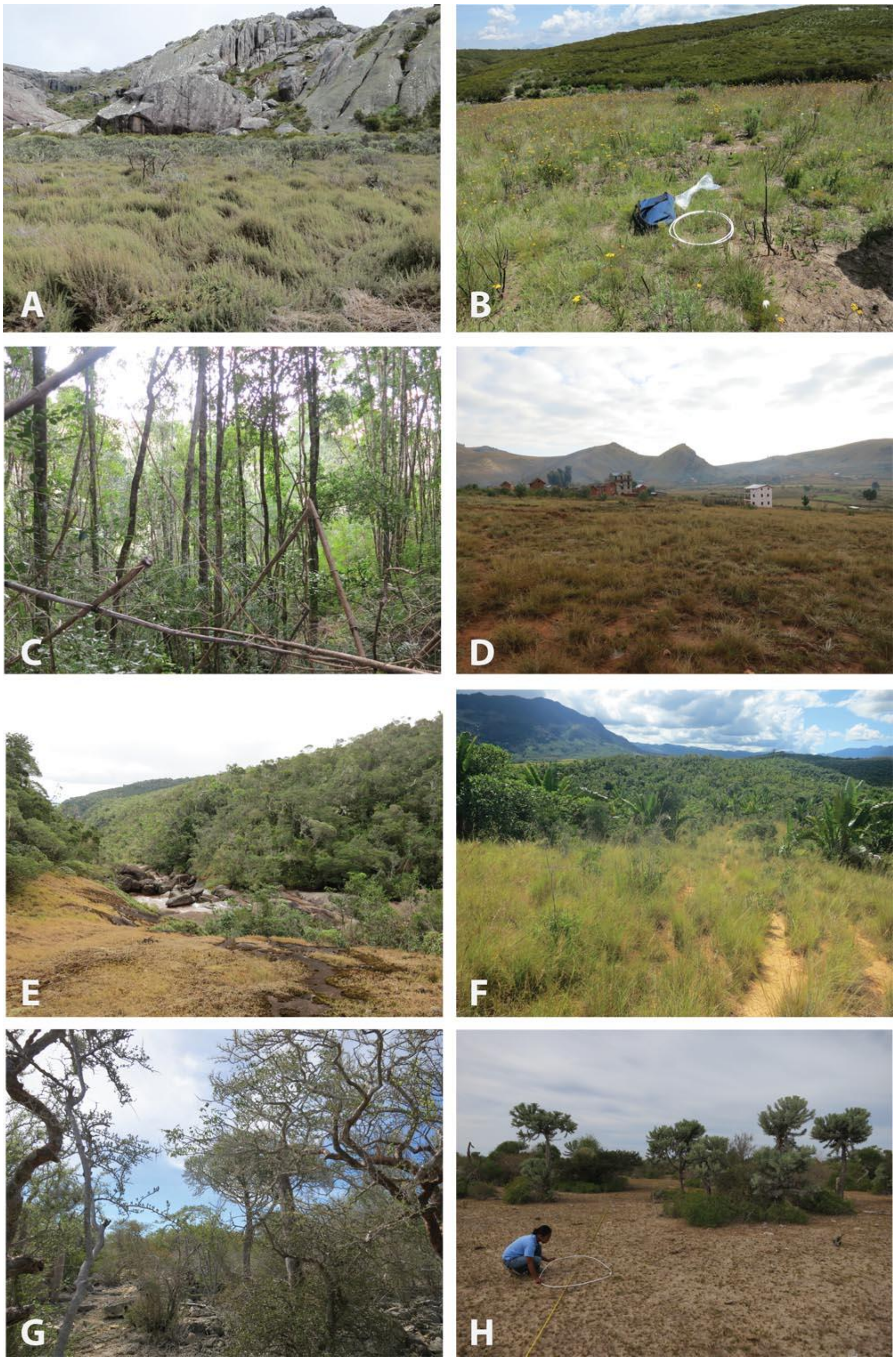

S3. Collection data and GenBank reference numbers for 206 Poaceae accessions used in this analysis. Species names and collection details are followed by acronyms of the herbaria where voucher specimens are preserved, fide Thiers, 2007. All sequences have been newly generated as part of this work and deposited at the European Nucleotide Archive with the following numbers: LN906638 to LN906796 (*matK*), LN907872 to LN908034 (*rbcL*), and LN908035 to LN908194 (*ndhF*).

Agrostis elliotii Hook. ex Scott-Elliot- Madagascar, Fianarantsoa, Anjomanakona: 2km east of Anjomanakona towards Ivato, 12km before Ivato, 20.6672° S, 47.1281° E Alt: 1547m, 27 February 2013, *Vorontsova, M.S.; Besnard, G.; Razanatsoa, J. & Rajaoanarison, R. 1037* (K,TAN).

Agrostis producta Pilg.- Tanzania, Arusha, Hanang, SITE 50 Hanang Protea hillside: Hanang Mountain, Udamaschek, Jerdom village valley, 4.4596° S, 35.3904° E Alt: 2772m, 13 June 2014, *Vorontsova, M.S.*; *Luke, W.R.Q.; Kimeu, J.M. & Kayombo, C. 1604* (EA,K,NHT).

Alloteropsis semialata (R.Br.) Hitchc.- Madagascar, Mahajanga, Ampitolova: ca 5km from Majunga to Antsianitia, 15.6661° S, 46.3708° E Alt: 25m, 14 February 2013, *Vorontsova, M.S.*; *Besnard, G.; Ralimanana, H. & Razanatsoa, J. 919* (K,TAN).

Anadelphia scyphofera Clayton- Tanzania, Rukwa, Mpanda, SITE 55 Uzondo grassland with Aristida humidicola: Uzondo Plateau S of Uvinza, road from Mpanda, 5.4839° S, 30.5300° E Alt: 1665m, 21 June 2014, *Vorontsova, M.S.*; *Luke, W.R.Q.; Kimeu, J.M. & Kayombo, C. 1652* (DSM,EA,FTI,K,NHT).

Andropogon africanus Franch.- Tanzania, Rukwa, Mpanda, SITE 55 Uzondo grassland with Aristida humidicola: Uzondo Plateau S of Uvinza, road from Mpanda, 5.4839° S, 30.5300° E Alt: 1665m, 21 June 2014, *Vorontsova, M.S.*; *Luke, W.R.Q.; Kimeu, J.M. & Kayombo, C. 1658* (EA,K,NHT).

Andropogon amethystinus Steud.- Tanzania, Arusha, Hanang, SITE 51 Hanang Protea ridge: Hanang Mountain, Udamaschek, Gabado village territory, 4.4524° S, 35.3875° E Alt: 3079m, 13 June 2014, *Vorontsova, M.S.*; *Luke, W.R.Q.; Kimeu, J.M. & Kayombo, C. 1606* (EA,K,NHT).

Andropogon andringitrensis (A.Camus) Voronts.- Madagascar, Fianarantsoa, SITE 9 XEROPHYTA HILL: Andringitra National Park, hills east of camp 3, 22.1890° S, 46.9073° E Alt: 2096m, 13 December 2013, *Vorontsova, M.S.*; *Linder, H.P.; Nanjarisoa, O.P. & Razanatsoa, J. 1260* (TAN).

Andropogon canaliculatus Schumach.- Tanzania, Rukwa, Mpanda, SITE 55 Uzondo grassland with Aristida humidicola: Uzondo Plateau S of Uvinza, road from Mpanda, 5.4839° S, 30.5300° E Alt: 1665m, 21 June 2014, *Vorontsova, M.S.*; *Luke, W.R.Q.; Kimeu, J.M. & Kayombo, C. 1662* (K).

Andropogon chrysostachyus Steud.- Tanzania, Arusha, Hanang, SITE 50 Hanang Protea hillside: Hanang Mountain, Udamaschek, Jerdom village valley, 4.4596° S, 35.3904° E Alt: 2772m, 13 June 2014, *Vorontsova, M.S.*; *Luke, W.R.Q.; Kimeu, J.M. & Kayombo, C. 1602* (EA,K,NHT).

Andropogon eucomus Nees- Madagascar, Antsiranana, Sambava, Marojejy National Park: path from camp 1 (camp Mantella) down towards the park entrance, ca 700 m before park entrance, 14.4555° S, 49.7932° E Alt: 193m, 19 October 2011, *Vorontsova, M.S.*; *Hall, R.C.; Besnard, G.; Rakotonasolo, F.; Rakotoarinivo, M. & Rajaonarison, R. 576* (K,TAN).

Andropogon ibityensis A.Camus- Madagascar, Fianarantsoa, Ambatoantrano: Itremo Protected Area, 20.5641° S, 46.5800° E Alt: 1723m, 14 March 2012, *Vorontsova, M.S.*; *Ratovonirina, G.; Rakotonasolo, F.; Rajaonarison, R.; Andriamampionina, R. & Rakotonirina, J.N. 754* (K,TAN).

Andropogon itremoensis Voronts.- Madagascar, Fianarantsoa, Ambatoantrano: Itremo Protected Area, 20.5641° S, 46.5800° E Alt: 1723m, 14 March 2012, *Vorontsova, M.S.; Ratovonirina, G.; Rakotonasolo, F.; Rajaonarison, R.; Andriamampionina, R. & Rakotonirina, J.N. 753* (K,TAN).

Andropogon trichozygus Baker- Madagascar, Fianarantsoa, Fianarantsoa II, Ranomafana: from Fianarantsoa to Ranomafana on RN25, just before the start of Ranomafana National Park, 21.2718° S, 47.3517° E Alt: 1182m, 28 October 2011, *Vorontsova, M.S.*; *Hall, R.C.; Besnard, G.; Ralimanana, H.; Randriamboavonjy, T. & Andriantiana, J. 619* (K,TAN).

Anthoxanthum nivale K.Schum.- Tanzania, Arusha, Hanang, SITE 52 Hanang Anthoxanthum nivale: Hanang Mountain, 2.2km before summit, 4.4468° S, 35.3892° E Alt: 3199m, 14 June 2014, *Vorontsova, M.S.*; *Luke, W.R.Q.; Kimeu, J.M. & Kayombo, C. 1618* (EA,FTI,K,NHT).

Aristida adoensis (R.Br.)- Tanzania, Arusha, Hanang, SITE 54 Hanang pasture: Hanang Mountain, Jerdom valley, Udamaschek village, 4.4975° S, 35.3959° E Alt: 1979m, 15 June 2014, *Vorontsova, M.S.*; *Luke, W.R.Q.; Kimeu, J.M. & Kayombo, C. 1643* (EA,FTI,K,NHT).

Aristida adscendsionis L.- Madagascar, Toliara, Betioky, Bezaha, 23.6833° S, 44.6333° E Alt: 175m, 11 December 1988, *Phillipson, P.B. 2859* (MO,P,TAN).

Aristida barbicollis Trin. & Rupr.- Madagascar, Toliara, along route 10 between Tongobory and Betioky, Alt: 100-300m, 14 February 1975, *Croat, T.B. 31212* (MO,P[P03346031],TAN).

Aristida cf. similis- Madagascar, Antsiranana, Ambilobe, Kelidada: 10.6 km N of Ambanja on E6, 13.4999° S, 48.7091° E Alt: 33m, 11 October 2011, *Vorontsova, M.S.*; *Hall, R.C.; Besnard, G.; Rakotonasolo, F.; Rakotoarinivo, M. & Rajaonarison, R. 346* (K,TAN).

Aristida humidicola- Tanzania, Rukwa, Mpanda, SITE 55 Uzondo grassland with Aristida humidicola: Uzondo Plateau S of Uvinza, road from Mpanda, 5.4839° S, 30.5300° E Alt: 1665m, 21 June 2014, *Vorontsova, M.S.*; *Luke, W.R.Q.; Kimeu, J.M. & Kayombo, C. 1651* (DSM,EA,FTI,K,NHT).

Aristida tenuissima A.Camus- Madagascar, Fianarantsoa, Ivato 1: 6 km from Ivato towards Ambatofinandrahana, 20.6575° S, 47.1614° E Alt: 1526m, 24 February 2013, *Vorontsova, M.S.*; *Besnard, G.; Razanatsoa, J. & Rajaoanarison, R. 991* (K,TAN).

Arundinaria- Madagascar, Fianarantsoa, Antsirakambiaty: Itremo Protected Area, 20.5950° S, 46.5631° E Alt: 1515m, 12 March 2012, *Vorontsova, M.S.*; *Ratovonirina, G.; Rakotonasolo, F.; Rajaonarison, R.; Andriamampionina, R. & Rakotonirina, J.N. 723* (K,TAN).

Arundinella nepalensis Trin.- Madagascar, Fianarantsoa, Ambalavao, Andringitra National Park: path from Belambo camp towards summit, 2.8 km from camp, 22.1458° S, 46.8911° E Alt: 1737m, 26 October 2011, *Vorontsova, M.S.*; *Hall, R.C.; Besnard, G.; Ralimanana, H.; Randriamboavonjy, T. & Andriantiana, J. 608* (K,TAN).

Bothriochloa insculpta (Hochst. ex A.Rich.) A.Camus- Tanzania, Arusha, Hanang, SITE 54 Hanang pasture: Hanang Mountain, Jerdom valley, Udamaschek village, 4.4975° S, 35.3959° E Alt: 1979m, 15 June 2014, *Vorontsova, M.S.*; *Luke, W.R.Q.; Kimeu, J.M. & Kayombo, C. 1637* (EA,FTI,K,NHT).

Bothriochloa sp.- Tanzania, Arusha, Hanang, SITE 53 Hanang Ehrharta forest: Hanang Mountain, Dahari forest, 4km S of Hanang peak, 4.4778° S, 35.3964° E Alt: 2143m, 14 June 2014, *Vorontsova, M.S.*; *Luke, W.R.Q.; Kimeu, J.M. & Kayombo, C. 1632* (K).

Brachiaria bemarivensis A.Camus- Madagascar, Mahajanga, Antsianitia Station Forestière: Antsianitia Station Forestière, 15.5783° S, 46.4153° E Alt: 20m, 15 February 2013, *Vorontsova, M.S.*; *Besnard, G.; Ralimanana, H. & Razanatsoa, J. 936* (K,TAN).

Brachiaria brizantha (A.Rich.) Stapf- Tanzania, Arusha, Hanang, SITE 54 Hanang pasture: Hanang Mountain, Jerdom valley, Udamaschek village, 4.4975° S, 35.3959° E Alt: 1979m, 15 June 2014, *Vorontsova, M.S.*; *Luke, W.R.Q.; Kimeu, J.M. & Kayombo, C. 1638* (EA,K,NHT).

Brachiaria dimorpha A.Camus- Madagascar, Fianarantsoa, SITE 3 POA STREAM: Andringitra National Park, near the road building shelter ca 500m below summit of Pic Boby, 22.1949° S, 46.8899° E Alt: 2470m, 12 December 2013, *Vorontsova, M.S.*; *Linder, H.P.; Nanjarisoa, O.P. & Razanatsoa, J. 1234* (TAN).

Brachiaria distachya (L.) Stapf- Madagascar, Mahajanga, Ambondromamy: 5-10km before Ambondromamy, before Kamoro bridge, 16.4333° S, 47.1500° E, 13 February 2013, *Vorontsova, M.S.*; *Besnard, G.; Ralimanana, H. & Razanatsoa, J. 915* (K,TAN).

Brachiaria epacridifolia (Stapf) A.Camus- Madagascar, Fianarantsoa, Antsirakambiaty: Itremo Protected Area, 20.5950° S, 46.5631° E Alt: 1515m, 12 March 2012, *Vorontsova, M.S.*; *Ratovonirina, G.; Rakotonasolo, F.; Rajaonarison, R.; Andriamampionina, R. & Rakotonirina, J.N. 722* (K,TAN).

Brachiaria hubbardii A.Camus- Mayotte, Grande Terre, Mamoudzou, prison de Majicavo, 26 December 2003, *Barthelat, F.; M'changama, M. 1256* (K[K000805479]).

Brachiaria humbertiana A.Camus- Madagascar, Toliara: SITE 23 Tsimanampesotsa dry forest; Lac Tsimanampesotsa National Park; near the viewpoint closest to the camping site, 24.0884° S, 43.7566° E Alt: 17m, 24 April 2014, *Vorontsova, M.S.*; *Besnard, G.; Razanatsoa, J.; Nanjarisoa, O.P. & Onjalalaina, G.E. 1378* (TAN).

Brachiaria reptans (L.) C.A.Gardner & C.E.Hubb.- Madagascar, Antsiranana, Ambanja, Ankify: mainland opposite Nosy Be, road from Ankify towards Doane, 13.5529° S, 48.3651° E Alt: 16m, 20 October 2011, *Vorontsova, M.S.*; *Hall, R.C.; Besnard, G.; Rakotonasolo, F.; Rakotoarinivo, M. & Rajaonarison, R. 583* (K,TAN).

Brachiaria semiundulata (Hochst. ex A.Rich.) Stapf- Tanzania, Arusha, Hanang, SITE 54 Hanang pasture: Hanang Mountain, Jerdom valley, Udamaschek village, 4.4975° S, 35.3959° E Alt: 1979m, 15 June 2014, *Vorontsova, M.S.*; *Luke, W.R.Q.; Kimeu, J.M. & Kayombo, C. 1645* (EA,FTI,K,NHT).

Brachiaria umbellata (Trin.) Clayton- Madagascar, Antsiranana, Ambilobe, Kelidada: 9.26km North of Ambanja on RN6 road, 13.5° S, 48.7° E Alt: 21m, 11 October 2011, *Hall, R.C.*; *Vorontsova, M.S.; Besnard, G.; Rakotonasolo, F. & Rakotoarinivo, M. 17* (K,TAN).

Brachypodium madagascariense A.Camus & H.Perrier- Madagascar, Fianarantsoa, Antsirakambiaty: Itremo Protected Area, 20.5950° S, 46.5631° E Alt: 1515m, 12 March 2012, *Vorontsova, M.S.*; *Ratovonirina, G.; Rakotonasolo, F.; Rajaonarison, R.; Andriamampionina, R. & Rakotonirina, J.N. 735* (K,TAN).

Bromus leptoclados Nees- Tanzania, Arusha, Hanang, SITE 51 Hanang Protea ridge: Hanang Mountain, Udamaschek, Gabado village territory, 4.4524° S, 35.3875° E Alt: 3079m, 13 June 2014, *Vorontsova, M.S.*; *Luke, W.R.Q.; Kimeu, J.M. & Kayombo, C. 1617* (K).

Cenchrus ciliaris L.- Madagascar, Toliara, Toliara: Antsapanimahavelona; A 17km au nord de Toliary, 23.3709° S, 43.8398° E Alt: 187m, 20 April 2014, *Nanjarisoa, O.P.*; *Vorontsova, M.S.; Besnard, G.; Razanatsoa, J. & Onjalalaina, G.E. 179* (K,TAN).

Chloris humbertiana A.Camus- Madagascar, Toliara, Beloha: Tranoroa; 25 km à la sortie du village d'Ampanihy pour Tranoroa, 24.6904° S, 44.9886° E Alt: 234m, 26 April 2014, *Nanjarisoa, O.P.*; *Vorontsova, M.S.; Besnard, G.; Razanatsoa, J. & Onjalalaina, G.E. 206* (K,TAN).

Chloris pycnothrix Trin.- Madagascar, Toamasina, Moramanga, Mantadia National Park: Sahanody Riviere, 12 km from Falierana park entrance; roadside at environs of camp 1, 18.8145° S, 48.4313° E Alt: 1004m, 8 October 2011, *Vorontsova, M.S.*; *Hall, R.C.; Besnard, G.; Rakotonasolo, F.; Rajaonarison, R. & Rakotondravelo, A. 313* (K,TAN).

Chrysopogon humbertianus A.Camus- Madagascar, Toliara: SITE 28 Chrysopogon humbertianus; ca 2 km from Tsihombe towards Cape Ste Marie, 25.5843° S, 45.1398° E Alt: 187m, 27 April 2014, *Vorontsova, M.S.*; *Besnard, G.; Razanatsoa, J.; Nanjarisoa, O.P. & Onjalalaina, G.E. 1413* (TAN).

Chrysopogon serrulatus Trin.- Madagascar, Mahajanga, Maevatanana, Berivotra area: road from Antananarive to Majunga, N of Maevatanana, 16.9597° S, 46.9213° E Alt: 207m, 10 October 2011, *Vorontsova, M.S.*; *Hall, R.C.; Besnard, G.; Rakotonasolo, F.; Rakotoarinivo, M. & Rajaonarison, R. 326* (K,TAN).

Craspedorhachys africana Benth.- Madagascar, Fianarantsoa, Ambatofinandrahana, Ankevo: Itremo, 20.6599° S, 46.5794° E Alt: 1682m, 13 March 2012, *Ratovonirina, G.*; *Vorontsova, M.S.; Rakotonasolo, F.; Rakotovao, A. & Rakotonirina, J.N. 188* (K,TAN).

Ctenium concinnum Nees- Madagascar, Fianarantsoa, Ambatofinandrahana, Ankevo: Itremo, 20.6598° S, 46.5794° E Alt: 1682m, 13 March 2012, *Ratovonirina, G.*; *Vorontsova, M.S.; Rakotonasolo, F.; Rakotovao, A. & Rakotonirina, J.N. 194* (K,TAN).

Cymbopogon caesius (Hook. & Arn.) Stapf- Madagascar, Fianarantsoa, Ambalavao, Andringitra National Park: path from Belambo camp towards summit, ca 500 m before first waterfall, 22.1456° S, 46.8917° E Alt: 1753m, 26 October 2011, *Vorontsova, M.S.*; *Hall, R.C.; Besnard, G.; Ralimanana, H.; Randriamboavonjy, T. & Andriantiana, J. 599* (K,TAN).

Cynodon nlemfuensis Vanderyst var. robustus Clayton & J.R.Harlan- Tanzania, Katavi region, Mpanda: Inyoga - Mpanda road Pt. 6, 6.4375° S, 31.6286° E Alt: 1316m, 19 June 2014, *Kimeu, J.M.*; *Vorontsova, M.S.; Luke, W.R.Q. & Kayombo, C. 221* (EA,FTI,K,NHT).

Cynodon radiatus Roth- Madagascar, Antsiranana, Ambanja, Ankify: road from Ankify towards Ambanja, 13.5529° S, 48.3663° E Alt: 10m, 2 November 2011, *Vorontsova, M.S.*; *Hall, R.C.; Besnard, G.; Ralimanana, H.; Randriamboavonjy, T. & Andriantiana, J. 715* (K,TAN).

Dactyloctenium capitatum A.Camus- Madagascar, Toliara: SITE 26 Euphorbia stenophylla pasture; 25 km N of Itampolo on the road from Tsimanampesotsa , 24.2226° S, 43.6857° E Alt: 8m, 25 April 2014, *Vorontsova, M.S.*; *Besnard, G.; Razanatsoa, J.; Nanjarisoa, O.P. & Onjalalaina, G.E. 1393* (TAN).

Decaryella madagascariensis A.Camus- Madagascar, Toliara: SITE 27 Decaryella; Belamoty; route from delta of the Linta to Ampanihy, ca 5 km after turn off from the coastal road inland , 25.0492° S, 44.3908° E Alt: 160m, 25 April 2014, *Vorontsova, M.S.*; *Besnard, G.; Razanatsoa, J.; Nanjarisoa, O.P. & Onjalalaina, G.E. 1398* (TAN).

Dichanthium aristatum (Poir.) C.E.Hubb.- Madagascar, Majunga, Boanamary: ca 2km from Majunga-Ankarafantsika road towards Boanamary, 15.7400° S, 46.4356° E Alt: 32m, 16 February 2013, *Vorontsova, M.S.*; *Besnard, G.; Ralimanana, H. & Razanatsoa, J. 949* (K,TAN).

Digitaria abyssinica (A.Rich.) Stapf- Madagascar, Fianarantsoa, Anjomanakona: 2km east of Anjomanakona towards Ivato, 12km before Ivato, 20.6672° S, 47.1281° E Alt: 1547m, 27 February 2013, *Vorontsova, M.S.*; *Besnard, G.; Razanatsoa, J. & Rajaoanarison, R. 1039* (K,TAN).

Digitaria ciliaris (Retz.) Koeler- Madagascar, Fianarantsoa, Ianasana 2: Itremo massif. 1.5km after Ianasana towards centre of the massif, 20.5778° S, 46.5744° E Alt: 1623m, 25 February 2013, *Vorontsova, M.S.*; *Besnard, G.; Razanatsoa, J. & Rajaoanarison, R. 1000* (K,TAN).

Digitaria longiflora (Retz.) Pers.- Madagascar, Fianarantsoa, Ianasana: Itremo Protected Area; stream crossing on road from Itremo village towards protected area, 20.5779° S, 46.5847° E Alt: 1562m, 13 March 2012, *Vorontsova, M.S.*; *Ratovonirina, G.; Rakotonasolo, F.; Rajaonarison, R.; Andriamampionina, R. & Rakotonirina, J.N. 745* (K,TAN).

Digitaria milanjiana (Rendle) Stapf- Kenya, Rift valley, Mbirikani ranch 1: Mbirikani ranch, near Ol Donyo Uasi lodge at the

plains., 2.5159° S, 37.7322° E Alt: 1149m, 18 December 2012, *Kimeu, J.M.*; *Vorontsova, M.S. & Luke, W.R.Q. 109* (EA,K).

Digitaria pseudodiagonalis Chiov.- Madagascar, Fianarantsoa, Andohantanimenaha 2: Itremo massif., 20.5217° S, 46.5664° E Alt: 1545m, 25 February 2013, *Vorontsova, M.S.*; *Besnard, G.; Razanatsoa, J. & Rajaoanarison, R. 1005* (K,TAN).

Digitaria velutina (Forssk.) P.Beauv.- Tanzania, Arusha, Hanang, SITE 54 Hanang pasture: Hanang Mountain, Jerdom valley, Udamaschek village, 4.4975° S, 35.3959° E Alt: 1979m, 15 June 2014, *Vorontsova, M.S.*; *Luke, W.R.Q.; Kimeu, J.M. & Kayombo, C. 1646* (EA,FTI,K,NHT).

Diheteropogon amplectens var. catangensis (Chiov.) Clayton- Tanzania, Tabora, Tabora, SITE 56 Uzondo campsite: Uzondo Plateau S of Uvinza, road from Mpanda, 5.4842° S, 32.5533° E Alt: 1670m, 21 June 2014, *Vorontsova, M.S.*; *Luke, W.R.Q.; Kimeu, J.M. & Kayombo, C. 1671* (EA,K).

Dimeria manongarivensis A.Camus- Madagascar, Antsiranana, Diana: SITE 38 Poecilostachys and Dimeria riverside; Reserve Speciale Manongarivo, Bekolosy river 2.3 km N of Ambalifary, 14.0455° S, 48.2964° E Alt: 1135m, 12 May 2014, *Vorontsova, M.S.*; *Onjalalaina, G.E. 1469* (P,TAN).

Echinochloa ugandensis Snowden & C.E.Hubb.- Madagascar, Fianarantsoa, Mananjary, Irondro: RN25 from Irondro to Ambolotara, 21.3868° S, 47.9386° E Alt: 82m, 4 November 2011, *Vorontsova, M.S.*; *Hall, R.C.; Besnard, G.; Ralimanana, H.; Randriamboavonjy, T. & Andriantiana, J. 706* (K,TAN).

Ehrharta erecta var. abyssinica (Hochst.) Pilg.- Tanzania, Arusha, Hanang, SITE 53 Hanang Ehrharta forest: Hanang Mountain, Dahari forest, 4km S of Hanang peak, 4.4778° S, 35.3964° E Alt: 2143m, 14 June 2014, *Vorontsova, M.S.*; *Luke, W.R.Q.; Kimeu, J.M. & Kayombo, C. 1625* (EA,K,NHT).

Eleusine multiflora Hochst. ex A.Rich.- Tanzania, Manyara region, Hanang district: Hanang government offices At the Hanang DCs office., 4.5151° S, 35.3889° E Alt: 1778m, 12 June 2014, *Kimeu, J.M.*; *Vorontsova, M.S.; Luke, W.R.Q. & Kayombo, C. 166* (EA,FTI,K,NHT).

Elionurus tristis Hack.- Madagascar, Fianarantsoa, Ambalavao, Andringitra National Park: path uphill from Andringitra park office and Namoly gate towards Belambo camp, ca 1 km away from park office, 22.1288° S, 46.9109° E Alt: 1588m, 25 October 2011, *Vorontsova, M.S.*; *Hall, R.C.; Besnard, G.; Ralimanana, H.; Randriamboavonjy, T. & Andriantiana, J. 589* (K,TAN).

Enneapogon cenchroides (Licht. ex Roem. & Schult.) C.E.Hubb.- Madagascar, Toliara, Sakaraha: Vineta; 5 km au sud du village de Sakaraha, 23.0500° S, 44.2500° E Alt: 399m, 20 April 2014, *Nanjarisoa, O.P.*; *Vorontsova, M.S.; Besnard, G.; Razanatsoa, J. & Onjalalaina, G.E. 174* (K,P,TAN).

Entolasia imbricata Stapf- Tanzania, Tabora, Tabora, SITE 56 Uzondo campsite: Uzondo Plateau S of Uvinza, road from Mpanda, 5.4842° S, 32.5533° E Alt: 1670m, 21 June 2014, *Vorontsova, M.S.*; *Luke, W.R.Q.; Kimeu, J.M. & Kayombo, C. 1675* (K).

Eragrostis boinensis A.Camus- Madagascar, Mahajanga, Antsianitia Station Forestière: Antsianitia Station Forestière, 15.5783° S, 46.4153° E Alt: 20m, 15 February 2013, *Vorontsova, M.S.*; *Besnard, G.; Ralimanana, H. & Razanatsoa, J. 935* (K,TAN).

Eragrostis canescens C.E.Hubb.- Tanzania, Rukwa, Mpanda, SITE 55 Uzondo grassland with Aristida humidicola: Uzondo Plateau S of Uvinza, road from Mpanda, 5.4839° S, 30.5300° E Alt: 1665m, 21 June 2014, *Vorontsova, M.S.*; *Luke, W.R.Q.; Kimeu, J.M. & Kayombo, C. 1660* (DSM,EA,FTI,K,NHT).

Eragrostis capuronii A.Camus- Madagascar, Toliara, Toliara: Antsarako; Aux environs des marais salant à l'ouest de Tuléar ville, route pour Mangily, 23.2920° S, 43.6439° E Alt: 24m, 21 April 2014, *Nanjarisoa, O.P.*; *Vorontsova, M.S.; Besnard, G.; Razanatsoa, J. & Onjalalaina, G.E. 186* (K,P,TAN).

Eragrostis chapelieri (Knuth.) Nees- Madagascar, Fianarantsoa, Vangaindrano, Monambondro: Sandravinany village, between Monambondro and Manantenina, 23.9853° S, 47.4638° E Alt: 45m, 30 October 2011, *Vorontsova, M.S.*; *Hall, R.C.; Besnard, G.; Ralimanana, H.; Randriamboavonjy, T. & Andriantiana, J. 639* (K,TAN).

Eragrostis dentifera Launert- Tanzania, Rukwa, Mpanda, SITE 55 Uzondo grassland with Aristida humidicola: Uzondo Plateau S of Uvinza, road from Mpanda, 5.4839° S, 30.5300° E Alt: 1665m, 21 June 2014, *Vorontsova, M.S.*; *Luke, W.R.Q.; Kimeu, J.M. & Kayombo, C. 1655* (DSM,EA,FTI,K,NHT).

Eragrostis gangetica (Roxb.) Steud.- Madagascar, Majunga, Station Forestière Marohogo 2: ca 3km on the track from Majunga-Ankarafantsika road, between Station Forestière Marohogo and Andradia, 15.7203° S, 46.4775° E Alt: 39m, 19 February 2013, *Vorontsova, M.S.*; *Besnard, G.; Ralimanana, H. & Razanatsoa, J. 968* (K,TAN).

Eragrostis humbertii A.Camus- Madagascar, Toliara: SITE 29 Eragrostis boinensis pasture; Ex-Bevazoa, ca 3 km from Cape Ste Marie towards the ANGAP office, village Bevzoa moved from here in the 1960s, 25.5840° S, 45.1421° E Alt: 186m, 27 April 2014, *Vorontsova, M.S.*; *Besnard, G.; Razanatsoa, J.; Nanjarisoa, O.P. & Onjalalaina, G.E. 1417* (TAN).

Eragrostis lateritica Bosser- Madagascar, Fianarantsoa, Vangaindrano, Vodivola: 2 km N of first ferry crossing at Vodivola,

23.6986° S, 47.5713° E Alt: 9m, 30 October 2011, *Vorontsova, M.S.*; *Hall, R.C.; Besnard, G.; Ralimanana, H.; Randriamboavonjy, T. & Andriantiana, J. 638* (K,TAN).

Eragrostis minor Host- Madagascar, Toliara: SITE 26 Euphorbia stenophylla pasture; 25 km N of Itampolo on the road from Tsimanampesotsa , 24.2226° S, 43.6857° E Alt: 8m, 25 April 2014, *Vorontsova, M.S.*; *Besnard, G.; Razanatsoa, J.; Nanjarisoa, O.P. & Onjalalaina, G.E. 1394* (TAN).

Eragrostis racemosa (Thunb.) Steud.- Tanzania, Arusha, Hanang, SITE 54 Hanang pasture: Hanang Mountain, Jerdom valley, Udamaschek village, 4.4975° S, 35.3959° E Alt: 1979m, 15 June 2014, *Vorontsova, M.S.*; *Luke, W.R.Q.; Kimeu, J.M. & Kayombo, C. 1644* (K).

Eragrostis schweinfurthii Chiov.- Tanzania, Arusha, Hanang, SITE 51 Hanang Protea ridge: Hanang Mountain, Udamaschek, Gabado village territory, 4.4524° S, 35.3875° E Alt: 3079m, 13 June 2014, *Vorontsova, M.S.*; *Luke, W.R.Q.; Kimeu, J.M. & Kayombo, C. 1610* (K).

Eragrostis tenella (L.) Roem. & Schultes var. insularis C.E.Hubb.- Madagascar, Toliara, Taolagnaro, Mandena: QIT Madagascar Minerals (QMM) head office, car park, NE of Taolagnaro, 24.9580° S, 47.0114° E Alt: 20m, 2 November 2011, *Vorontsova, M.S.*; *Hall, R.C.; Besnard, G.; Ralimanana, H.; Randriamboavonjy, T. & Andriantiana, J. 699* (K,TAN).

Eragrostis tenuifolia L.- Madagascar, Toamasina, Moramanga, Mantadia National Park: Sahanody Riviere, 12 km from Falierana park entrance; roadside at environs of camp 1, 18.8145° S, 48.4313° E Alt: 1004m, 8 October 2011, *Vorontsova, M.S.*; *Hall, R.C.; Besnard, G.; Rakotonasolo, F.; Rajaonarison, R. & Rakotondravelo, A. 312* (K,TAN).

Exotheca abyssinica (Hochst. ex A.Rich.) Andersson- Tanzania, Arusha, Hanang, SITE 50 Hanang Protea hillside: Hanang Mountain, Udamaschek, Jerdom village valley, 4.4596° S, 35.3904° E Alt: 2772m, 13 June 2014, *Vorontsova, M.S.*; *Luke, W.R.Q.; Kimeu, J.M. & Kayombo, C. 1599* (DSM,FTI,K,NHT).

Festuca abyssinica A.Rich.- Tanzania, Arusha, Hanang, SITE 50 Hanang Protea hillside: Hanang Mountain, Udamaschek, Jerdom village valley, 4.4596° S, 35.3904° E Alt: 2772m, 13 June 2014, *Vorontsova, M.S.*; *Luke, W.R.Q.; Kimeu, J.M. & Kayombo, C. 1601* (K).

Festuca camusiana St.-Yves- Madagascar, Fianarantsoa, Fianarantsoa II, Ranomafana: RN25, 15 km from junction towards Ranomafana National Park, 21.2823° S, 47.3206° E Alt: 1217m, 28 October 2011, *Vorontsova, M.S.*; *Hall, R.C.; Besnard, G.; Ralimanana, H.; Randriamboavonjy, T. & Andriantiana, J. 617* (K,TAN).

Festuca obturbans St.-Yves- Tanzania, Manyara region, Mt Hanang: Tourists track from Kateshi side Pt. 5 At Erica zone and following Udamascheki toursts route., 4.4515° S, 35.3877° E Alt: 3064m, 13 June 2014, *Kimeu, J.M.*; *Vorontsova, M.S.; Luke, W.R.Q. & Kayombo, C. 173* (EA,FTI,K,NHT).

Helictotrichon elongatum (Hochst. ex A.Rich.) C.E.Hubb.- Madagascar, Fianarantsoa, Andringitra camp 1: Andringitra National Park; below camp 1 Belambo, 22.1336° S, 46.8908° E Alt: 1569m, 27 November 2013, *Vorontsova, M.S.*; *Cleave, M.; Nanjarisoa, O.P. & Rakotonasolo, F. 1215* (TAN).

Heteropogon contortus (L.) P.Beauv. ex Roem. & Schult.- Madagascar, Mahajanga, Maevatanana, Berivotra area: road from Antananarive to Majunga, N of Maevatanana, 16.9597° S, 46.9213° E Alt: 207m, 10 October 2011, *Vorontsova, M.S.*; *Hall, R.C.; Besnard, G.; Rakotonasolo, F.; Rakotoarinivo, M. & Rajaonarison, R. 327* (K,TAN).

Heteropogon melanocarpus Benth.- Madagascar, Fianarantsoa, Ambatofinandrahana: Itremo, Antsirakambiaty; A l'entrée de la première forêt galérie d'Antsirakambiaty, 20.5961° S, 46.5629° E Alt: 1520m, 18 February 2014, *Nanjarisoa, O.P.*; *Ralimanana, H. & Razafindraibe, A. 139* (K,TAN).

Hickelia madagascariensis A.Camus- Madagascar, Fianarantsoa, Antsirakambiaty: Itremo Protected Area, 20.5950° S, 46.5631° E Alt: 1515m, 12 March 2012, *Vorontsova, M.S.*; *Ratovonirina, G.; Rakotonasolo, F.; Rajaonarison, R.; Andriamampionina, R. & Rakotonirina, J.N. 724* (K,TAN).

Humbertochloa bambusiuscula A.Camus & Stapf- Madagascar, Majunga, Ambalakida: ca 5km before Andradia on the track from Majunga-Ankarafantsika road towards Anjohibe cave, 15.7081° S, 46.5275° E Alt: 27m, 16 February 2013, *Vorontsova, M.S.*; *Besnard, G.; Ralimanana, H. & Razanatsoa, J. 956* (K,TAN).

Hyparrhenia anamesa Clayton- Tanzania, Arusha, Hanang, SITE 54 Hanang pasture: Hanang Mountain, Jerdom valley, Udamaschek village, 4.4975° S, 35.3959° E Alt: 1979m, 15 June 2014, *Vorontsova, M.S.*; *Luke, W.R.Q.; Kimeu, J.M. & Kayombo, C. 1640* (EA,K,NHT).

Hyparrhenia collina (Pilg.) Stapf- Tanzania, Arusha, Hanang, SITE 54 Hanang pasture: Hanang Mountain, Jerdom valley, Udamaschek village, 4.4975° S, 35.3959° E Alt: 1979m, 15 June 2014, *Vorontsova, M.S.*; *Luke, W.R.Q.; Kimeu, J.M. & Kayombo, C. 1639* (EA,K,NHT).

Hyparrhenia cymbaria (L.) Stapf- Tanzania, Arusha, Hanang, SITE 53 Hanang Ehrharta forest: Hanang Mountain, Dahari forest, 4km S of Hanang peak, 4.4778° S, 35.3964° E Alt: 2143m, 14 June 2014, *Vorontsova, M.S.*; *Luke, W.R.Q.; Kimeu, J.M. & Kayombo, C. 1628* (EA,FTI,K,NHT).

Hyparrhenia newtonii (Hack.) Stapf- Madagascar, Fianarantsoa, Antsirakambiaty: Itremo Protected Area, 20.5950° S, 46.5631° E Alt: 1515m, 12 March 2012, *Vorontsova, M.S.*; *Ratovonirina, G.; Rakotonasolo, F.; Rajaonarison, R.;*

Andriamampionina, R. & Rakotonirina, J.N. 721 (K,TAN).

Hyparrhenia rufa (Nees) Stapf- Madagascar, Mahajanga, Maevatanana, Berivotra area: road from Antananarive to Majunga, N of Maevatanana, 16.9597° S, 46.9213° E Alt: 207m, 10 October 2011, *Vorontsova, M.S.*; *Hall, R.C.; Besnard, G.; Rakotonasolo, F.; Rakotoarinivo, M. & Rajaonarison, R. 328* (K,TAN).

Hyparrhenia schimperi (Hochst. ex A.Rich.) Andersson ex Stapf- Madagascar, Fianarantsoa, Antsirakambiaty: Itremo Protected Area, 20.5950° S, 46.5631° E Alt: 1515m, 12 March 2012, *Vorontsova, M.S.*; *Ratovonirina, G.; Rakotonasolo, F.; Rajaonarison, R.; Andriamampionina, R. & Rakotonirina, J.N. 720* (K).

Isachne mauritiana Knuth.- Madagascar, Toamasina, Moramanga, Mantadia National Park: Sahanody Riviere, 12 km from Falierana park entrance. Trail from camp 1 uphill, 18.8145° S, 48.4341° E Alt: 1072m, 8 October 2011, *Vorontsova, M.S.*; *Hall, R.C.; Besnard, G.; Rakotonasolo, F.; Rajaonarison, R. & Rakotondravelo, A. 308* (K,TAN).

Isalus isalensis (A.Camus) Phipps- Madagascar, Fianarantsoa, Ihorombe: near SITE 22 Trachypogon grassland outside Isalo; ca 5 km from Ranohira towards Llakaka, 22.6267° S, 45.3495° E Alt: 819m, 20 April 2014, *Vorontsova, M.S.*; *Besnard, G.; Razanatsoa, J.; Nanjarisoa, O.P. & Onjalalaina, G.E. 1370* (TAN).

Ischaemum koleostachys Hack.- Madagascar, Toliara, SITE 14 NAMAZA STREAM BANK: Isalo National Park, Namaza River bank above Namaza camp, 22.5389° S, 45.3785° E Alt: 780m, 16 December 2013, *Vorontsova, M.S.*; *Linder, H.P.; Nanjarisoa, O.P. & Razanatsoa, J. 1299* (TAN).

Lecomtella madagascariensis A.Camus- Madagascar, Fianarantsoa, Ambalavao, Andringitra National Park: path from Belambo camp towards summit, 2.8 km from camp, 22.1458° S, 46.8911° E Alt: 1737m, 26 October 2011, *Vorontsova, M.S.*; *Hall, R.C.; Besnard, G.; Ralimanana, H.; Randriamboavonjy, T. & Andriantiana, J. 603* (K,TAN).

Leptaspis cochleata Thwaites- Madagascar, Antsiranana, Vohemar, Daraina Protected Area: Binara mountain, 13.3° S, 49.6° E Alt: 564m, 13 October 2011, *Hall, R.C.*; *Vorontsova, M.S.; Besnard, G.; Rakotonasolo, F. & Rakotoarinivo, M. 24* (K,TAN).

Lepturus anadabolavensis A.Camus- Madagascar, Toliara: Tsimanampetsotsa; Près du campement de Tsiamaso dans le parc national de Tsimanampetsotsa, 24.0424° S, 43.7640° E Alt: 2m, 24 April 2014, *Nanjarisoa, O.P.*; *Vorontsova, M.S.; Besnard, G.; Razanatsoa, J. & Onjalalaina, G.E. 203* (K,TAN).

Lepturus humbertianus A.Camus- Madagascar, Toliara: SITE 27 Decaryella; Belamoty; route from delta of the Linta to Ampanihy, ca 5 km after turn off from the coastal road inland , 25.0492° S, 44.3908° E Alt: 160m, 25 April 2014, *Vorontsova, M.S.*; *Besnard, G.; Razanatsoa, J.; Nanjarisoa, O.P. & Onjalalaina, G.E. 1403* (P,TAN).

Lepturus radicans (Steud.) A.Camus- Madagascar, Mahajanga, Antsianitia Station Forestière: Antsianitia Station Forestière, 15.5783° S, 46.4153° E Alt: 20m, 15 February 2013, *Vorontsova, M.S.*; *Besnard, G.; Ralimanana, H. & Razanatsoa, J. 934* (K,TAN).

Loudetia lanata (Stent & J.M.Rattray) C.E.Hubb.- Tanzania, Rukwa, Mpanda, SITE 55 Uzondo grassland with Aristida humidicola: Uzondo Plateau S of Uvinza, road from Mpanda, 5.4839° S, 30.5300° E Alt: 1665m, 21 June 2014, *Vorontsova, M.S.*; *Luke, W.R.Q.; Kimeu, J.M. & Kayombo, C. 1654* (K).

Loudetia simplex (Nees) C.E.Hubb.- Madagascar, Fianarantsoa, Ambalavao, Andringitra National Park: path uphill from Andringitra park office and Namoly gate towards Belambo camp, just before camp, 22.1353° S, 46.8909° E Alt: 1624m, 25 October 2011, *Vorontsova, M.S.*; *Hall, R.C.; Besnard, G.; Ralimanana, H.; Randriamboavonjy, T. & Andriantiana, J. 595* (K,TAN).

Megathyrsus maximus (Jacq.) B.K.Simon & Jacobs- Madagascar, Antsiranana, Ambilobe, Kelidada: 9.47 km N of Ambanja on E6, 13.5065° S, 48.7011° E Alt: 30m, 11 October 2011, *Vorontsova, M.S.*; *Hall, R.C.; Besnard, G.; Rakotonasolo, F.; Rakotoarinivo, M. & Rajaonarison, R. 345* (K,TAN).

Merxmuellera ambalavaoensis (A.Camus) Conert- Madagascar, Fianarantsoa, SITE 3 POA STREAM: Andringitra National Park, near the road building shelter ca 500m below summit of Pic Boby, 22.1949° S, 46.8899° E Alt: 2470m, 12 December 2013, *Vorontsova, M.S.*; *Linder, H.P.; Nanjarisoa, O.P. & Razanatsoa, J. 1235* (TAN).

Microchloa kunthii Desv.- Madagascar, Fianarantsoa, Ambatoantrano: Itremo Protected Area, 20.5641° S, 46.5800° E Alt: 1723m, 14 March 2012, *Vorontsova, M.S.*; *Ratovonirina, G.; Rakotonasolo, F.; Rajaonarison, R.; Andriamampionina, R. & Rakotonirina, J.N. 746* (K,TAN).

Monocymbium ceresiiforme (Nees) Stapf- Tanzania, Tabora, Tabora, SITE 56 Uzondo campsite: Uzondo Plateau S of Uvinza, road from Mpanda, 5.4842° S, 32.5533° E Alt: 1670m, 21 June 2014, *Vorontsova, M.S.*; *Luke, W.R.Q.; Kimeu, J.M. & Kayombo, C. 1679* (DSM,EA,FTI,K,NHT).

Nastus aristatus A.Camus- Madagascar, Antsiranana, Diana: SITE 36 Nastus aristatus forest; Reserve Speciale Manongarivo, Bekolosy forest 2 km N of Ambalifary, 14.0478° S, 48.2958° E Alt: 1055m, 12 May 2014, *Vorontsova, M.S.*; *Onjalalaina, G.E. 1464* (TAN).

Neostapfiella perrieri A.Camus- Madagascar, Antsiranana, Diana: between Reserve Speciale Manongarivo and RN6, 2km west of Antsambalahy village, 14.0465° S, 48.2168° E Alt: 138m, 13 May 2014, *Vorontsova, M.S.*; *Razanatsoa, J. &*

Nanjarisoa, O.P. 1486 (TAN).

Olyra latifolia L.- Guyana, July 2012, *Besnard, G. s.n.* (K).

Oplismenus burmannii (Retz.) P.Beauv.- Madagascar, Antsiranana, Sambava, Marojejy National Park: 151m North of Camp Mantella on the path to Cascade de Humbert, 14.4362° S, 49.7755° E Alt: 477m, 18 October 2011, *Hall, R.C.*; *Vorontsova, M.S.; Besnard, G.; Rakotonasolo, F. & Rakotoarinivo, M. 41* (K,TAN).

Oplismenus compositus (L.) P.Beauv.- Madagascar, Ankazobe, Ankazobe: ca 30 km N of Ankazobe, between Ankazobe and Manerinerina, 18.0336° S, 47.1747° E, 21 February 2013, *Vorontsova, M.S.*; *Besnard, G.; Ralimanana, H. & Razanatsoa, J. 984* (K,TAN).

Oplismenus flavicomus Mez- Madagascar, Fianarantsoa, Antsirakambiaty: Itremo Protected Area, 20.5950° S, 46.5631° E Alt: 1515m, 12 March 2012, *Vorontsova, M.S.*; *Ratovonirina, G.; Rakotonasolo, F.; Rajaonarison, R.; Andriamampionina, R. & Rakotonirina, J.N. 737* (K,TAN).

Oplismenus hirtellus (L.) P.Beauv.- Madagascar, Antsiranana, Sambava, Marojejy National Park: near path between Camp Mantella and park entrance about 1km from the park entrance., 14.5° S, 49.8° E Alt: 248m, 19 October 2011, *Hall, R.C.*; *Vorontsova, M.S.; Besnard, G.; Rakotonasolo, F. & Rakotoarinivo, M. 42* (K,TAN).

Oplismenus sp. nov.- Madagascar, Antsiranana, Diana: SITE 38 Poecilostachys and Dimeria riverside; Reserve Speciale Manongarivo, Bekolosy river 2.3 km N of Ambalifary, 14.0455° S, 48.2964° E Alt: 1135m, 12 May 2014, *Vorontsova, M.S.*; *Onjalalaina, G.E. 1468* (P,TAN).

Panicum andringitrense A.Camus- Madagascar, Fianarantsoa, Ambalavao, Andringitra National Park: path uphill from Andringitra park office and Namoly gate towards Belambo camp, 4.4 km away from park office, 22.1311° S, 46.8921° E Alt: 1574m, 25 October 2011, *Vorontsova, M.S.*; *Hall, R.C.; Besnard, G.; Ralimanana, H.; Randriamboavonjy, T. & Andriantiana, J. 591* (K,TAN).

Panicum brevifolium L.- Madagascar, Toamasina, Moramanga, Mantadia National Park: Camp 1: Sahanody Riviere, 12 km from Falierana park entrance, 18.8135° S, 48.4301° E Alt: 940m, 7 October 2011, *Vorontsova, M.S.*; *Hall, R.C.; Besnard, G.; Rakotonasolo, F.; Rajaonarison, R. & Rakotondravelo, A. 304* (K,TAN).

Panicum cinctum Hack.- Madagascar, Fianarantsoa, Soatsihotapaka: Itremo massif., 20.5122° S, 46.5786° E Alt: 1643m, 26 February 2013, *Vorontsova, M.S.*; *Besnard, G.; Razanatsoa, J. & Rajaoanarison, R. 1027* (K,TAN).

Panicum cupressifolium A.Camus- Madagascar, Fianarantsoa, SITE 3 POA STREAM: Andringitra National Park, near the road building shelter ca 500m below summit of Imarivolanitra (formally Pic Boby), 22.1949° S, 46.8899° E Alt: 2470m, 12 December 2013, *Vorontsova, M.S.*; *Linder, H.P.; Nanjarisoa, O.P. & Razanatsoa, J. 1233* (TAN).

Panicum flacciflorum Stapf- Tanzania, Katavi region, Mpanda: Mpanda - Uvinza road Pt. 5 Uzondo plateaue., 5.4997° S, 30.5306° E Alt: 1261m, 20 June 2014, *Kimeu, J.M.*; *Vorontsova, M.S.; Luke, W.R.Q. & Kayombo, C. 236* (EA,FTI,K,NHT).

Panicum hymeniochilum Nees- Madagascar, Toamasina, Moramanga, Mantadia National Park: Sahanody Riviere, 12 km from Falierana park entrance. Streamside path from camp 1, 18.8102° S, 48.4277° E Alt: 960m, 8 October 2011, *Vorontsova, M.S.*; *Hall, R.C.; Besnard, G.; Rakotonasolo, F.; Rajaonarison, R. & Rakotondravelo, A. 323* (K,TAN).

Panicum malacotrichum Steud.- Madagascar, Antsiranana, Ambilobe, Kelidada: 9.47km North of Ambanja on RN6 road, 13.5° S, 48.7° E Alt: 30m, 11 October 2011, *Hall, R.C.*; *Vorontsova, M.S.; Besnard, G.; Rakotonasolo, F. & Rakotoarinivo, M. 19* (K,TAN).

Panicum parvifolium Lam.- Madagascar, Antsiranana, Ambilobe, Kelidada: 9.26km North of Ambanja on RN6 road., 13.5° S, 48.7° E Alt: 21m, 11 October 2011, *Hall, R.C.*; *Vorontsova, M.S.; Besnard, G.; Rakotonasolo, F. & Rakotoarinivo, M. 16* (K,TAN).

Panicum perrieri A.Camus- Madagascar, Fianarantsoa, Ambatoantrano: Itremo Protected Area, 20.5641° S, 46.5800° E Alt: 1723m, 14 March 2012, *Vorontsova, M.S.*; *Ratovonirina, G.; Rakotonasolo, F.; Rajaonarison, R.; Andriamampionina, R. & Rakotonirina, J.N. 749* (K,TAN).

Panicum pseudowoeltzkowii A.Camus- Madagascar, Mahajanga, Antsianitia Station Forestière: Antsianitia Station Forestière, 15.5783° S, 46.4153° E Alt: 20m, 15 February 2013, *Vorontsova, M.S.*; *Besnard, G.; Ralimanana, H. & Razanatsoa, J. 937* (K,TAN).

Panicum sp. nov.- Madagascar, Fianarantsoa, Diavolana: Andringitra National Park; Diavolana, 6 km from camp 2 towards camp 4, 22.1370° S, 46.8666° E Alt: 2149m, 29 November 2013, *Vorontsova, M.S.*; *Cleave, M.; Nanjarisoa, O.P. & Rakotonasolo, F. 1218* (TAN).

Panicum spergulifolium A.Camus- Madagascar, Fianarantsoa, SITE 7 SPONGE: Andringitra National Park, basin east of camp 3, 22.1922° S, 46.9056° E Alt: 2054m, 13 December 2013, *Vorontsova, M.S.*; *Linder, H.P.; Nanjarisoa, O.P. & Razanatsoa, J. 1250* (TAN).

Panicum subhystrix A.Camus- Madagascar, Fianarantsoa, Ambatoantrano: Itremo Protected Area, 20.5641° S, 46.5800° E

Alt: 1723m, 14 March 2012, *Vorontsova, M.S.*; *Ratovonirina, G.; Rakotonasolo, F.; Rajaonarison, R.; Andriamampionina, R. & Rakotonirina, J.N. 748* (K,TAN).

Panicum trichocladum K.Schum.- Madagascar, Fianarantsoa, Mananjary, Irondro: RN25 from Irondro to Ambolotara, 21.3868° S, 47.9386° E Alt: 82m, 4 November 2011, *Vorontsova, M.S.*; *Hall, R.C.; Besnard, G.; Ralimanana, H.; Randriamboavonjy, T. & Andriantiana, J. 705* (K,TAN).

Panicum vohitrense A.Camus- Madagascar, Antsiranana, Diana: SITE 37 Panicum vohitrense granite wall; Reserve Speciale Manongarivo, Bekolosy forest 2 km N of Ambalifary, 14.0473° S, 48.2960° E Alt: 1075m, 12 May 2014, *Vorontsova, M.S.*; *Onjalalaina, G.E. 1466* (TAN).

Panicum wiehei Renvoize- Tanzania, Arusha, Hanang, SITE 53 Hanang Ehrharta forest: Hanang Mountain, Dahari forest, 4km S of Hanang peak, 4.4778° S, 35.3964° E Alt: 2143m, 14 June 2014, *Vorontsova, M.S.*; *Luke, W.R.Q.; Kimeu, J.M. & Kayombo, C. 1626* (EA,FTI,K,NHT).

Paspalum conjugatum L.- Madagascar, Toamasina, Moramanga, Mantadia National Park: Sahanody Riviere, 12 km from Falierana park entrance; roadside at environs of camp 1, 18.8135° S, 48.4301° E Alt: 940m, 8 October 2011, *Vorontsova, M.S.*; *Hall, R.C.; Besnard, G.; Rakotonasolo, F.; Rajaonarison, R. & Rakotondravelo, A. 316* (K,TAN).

Paspalum scrobiculatum L.- Madagascar, Antsiranana, Sambava, Marojejy National Park: path from camp 1 down towards the park entrance, ca 700 m before park entrance, 14.4634° S, 49.7971° E Alt: 175m, 19 October 2011, *Vorontsova, M.S.*; *Hall, R.C.; Besnard, G.; Rakotonasolo, F.; Rakotoarinivo, M. & Rajaonarison, R. 577* (K,TAN).

Paspalum vaginatum Sw.- Madagascar, Mahajanga, Antsianitia coast: behind Antsianitia village, 15.5697° S, 46.4156° E Alt: 16m, 14 February 2013, *Vorontsova, M.S.*; *Besnard, G.; Ralimanana, H. & Razanatsoa, J. 927* (K,TAN).

Pennisetum polystachion (L.) Schult.- Madagascar, Mahajanga, Maevatanana, Betsiboka: N of bridge across Betsiboka river, 16.8926° S, 46.9687° E Alt: 172m, 10 October 2011, *Vorontsova, M.S.*; *Hall, R.C.; Besnard, G.; Rakotonasolo, F.; Rakotoarinivo, M. & Rajaonarison, R. 334* (K,TAN).

Pentameris borussica (K.Schum.) Galley & H.P.Linder- Tanzania, Arusha, Hanang, SITE 51 Hanang Protea ridge: Hanang Mountain, Udamaschek, Gabado village territory, 4.4524° S, 35.3875° E Alt: 3079m, 13 June 2014, *Vorontsova, M.S.*; *Luke, W.R.Q.; Kimeu, J.M. & Kayombo, C. 1609* (K).

Phragmites mauritianus- Madagascar, Mahajanga, Maevatanana, Betsiboka: N of bridge across Betsiboka river, 16.8926° S, 46.9687° E Alt: 172m, 10 October 2011, *Vorontsova, M.S.*; *Hall, R.C.; Besnard, G.; Rakotonasolo, F.; Rakotoarinivo, M. & Rajaonarison, R. 332* (K,TAN).

Poa leptoclada Hochst. ex A.Rich.- Tanzania, Arusha, Hanang, SITE 51 Hanang Protea ridge: Hanang Mountain, Udamaschek, Gabado village territory, 4.4524° S, 35.3875° E Alt: 3079m, 13 June 2014, *Vorontsova, M.S.*; *Luke, W.R.Q.; Kimeu, J.M. & Kayombo, C. 1611* (K,NHT).

Poa perrieri A.Camus- Madagascar, Fianarantsoa, SITE 3 POA STREAM: Andringitra National Park, near the road building shelter ca 500m below summit of Imarivolanitra (formally Pic Boby), 22.1949° S, 46.8899° E Alt: 2470m, 12 December 2013, *Vorontsova, M.S.*; *Linder, H.P.; Nanjarisoa, O.P. & Razanatsoa, J. 1232* (TAN).

Saccharum cf. viguieri- Madagascar, Fianarantsoa, Ambalavao, Andringitra National Park: path from Belambo camp towards summit, 2.8 km from camp, 22.1458° S, 46.8911° E Alt: 1737m, 26 October 2011, *Vorontsova, M.S.*; *Hall, R.C.; Besnard, G.; Ralimanana, H.; Randriamboavonjy, T. & Andriantiana, J. 604* (K,TAN).

Saccharum perrieri (A.Camus) Clayton- Madagascar, Fianarantsoa, Ambatofinandrahana: Itremo, Antsirakambiaty; Juste au dessus de la forêt galérie, 20.5961° S, 46.5630° E Alt: 1519m, 18 February 2014, *Nanjarisoa, O.P.*; *Ralimanana, H. & Razafindraibe, A. 140* (K,TAN).

Saccharum viguieri (A.Camus) Clayton- Madagascar, Fianarantsoa, SITE 6 SACCHARUM HILLSIDE: Andringitra National Park, path above camp 3 to pic Boby, 22.1927° S, 46.8998° E Alt: 2261m, 13 December 2013, *Vorontsova, M.S.*; *Linder, H.P.; Nanjarisoa, O.P. & Razanatsoa, J. 1248* (TAN).

Sacciolepis curvata (L.) Chase- Madagascar, Antsiranana, Vohemar, Daraina Protected Area: Binara mountain; path towards Binara summit, 13.2572° S, 49.6135° E Alt: 419m, 13 October 2011, *Vorontsova, M.S.*; *Hall, R.C.; Besnard, G.; Rakotonasolo, F.; Rakotoarinivo, M.; Rajaonarison, R. & Léopold 394* (K,TAN).

Schizachirium brevifolium (Sw.) Buse- Madagascar, Fianarantsoa, Ambatofinandrahana: Antsahakely, 20.5621° S, 46.9309° E Alt: 1534m, 15 March 2012, *Ratovonirina, G.; Vorontsova, M.S.; Rakotonasolo, F.; Rakotovao, A. & Rakotonirina, J.N. 199* (K,TAN).

Schizachyrium sanguineum (Retz.) Alston- Madagascar, Fianarantsoa, Ambatofinandrahana, Ambazimba: Itremo, along the RN 35, 20.5666° S, 46.5971° E Alt: 1382m, 13 March 2012, *Ratovonirina, G.*; *Vorontsova, M.S.; Rakotonasolo, F.; Rakotovao, A. & Rakotonirina, J.N. 180* (K,TAN).

Sclerodactylon macrostachyum (Benth.) A.Camus- Madagascar, Toliara, Toliara II: 50m au nord de l'aéroport de Tuléar, bord de RN7, 23.3943° S, 43.7342° E Alt: 3m, 22 April 2014, *Nanjarisoa, O.P.*; *Vorontsova, M.S.; Besnard, G.; Razanatsoa, J. & Onjalalaina, G.E. 198* (K,N/A,TAN).

Setaria humbertiana A.Camus- Madagascar, Toliara: SITE 28 Chrysopogon humbertianus; ca 2 km from Tsihombe towards Cape Ste Marie, 25.5843° S, 45.1398° E Alt: 187m, 27 April 2014, *Vorontsova, M.S.*; *Besnard, G.; Razanatsoa, J.; Nanjarisoa, O.P. & Onjalalaina, G.E. 1414* (TAN).

Setaria incrassata (Hochst.) Hack.- Tanzania, Arusha, Hanang, SITE 54 Hanang pasture: Hanang Mountain, Jerdom valley, Udamaschek village, 4.4975° S, 35.3959° E Alt: 1979m, 15 June 2014, *Vorontsova, M.S.*; *Luke, W.R.Q.; Kimeu, J.M. & Kayombo, C. 1641* (EA,FTI,K,NHT).

Setaria madecassa A.Camus- Madagascar, Majunga, Ankarafantsika: 10-20 km SW after Ankarafantsika NP, 16.3600° S, 46.9075° E Alt: 158m, 19 February 2013, *Vorontsova, M.S.*; *Besnard, G.; Ralimanana, H. & Razanatsoa, J. 979* (K,TAN).

Setaria megaphylla (Steud.) T.Durand & Schinz- Madagascar, Toamasina, Moramanga, Mantadia National Park: Sahanody Riviere, 12 km from Falierana park entrance; ca 15 m from the road near camp 1, 18.8153° S, 48.4324° E Alt: 998m, 8 October 2011, *Vorontsova, M.S.*; *Hall, R.C.; Besnard, G.; Rakotonasolo, F.; Rajaonarison, R. & Rakotondravelo, A. 310* (K,TAN).

Setaria sphacelata (Schumach.) Stapf & C.E.Hubb. ex Moss- Madagascar, Fianarantsoa, Ambatofinandrahana, Ambatomenaloha: Itremo, 20.6191° S, 46.5583° E Alt: 1686m, 13 March 2012, *Ratovonirina, G.*; *Vorontsova, M.S.; Rakotonasolo, F.; Rakotovao, A. & Rakotonirina, J.N. 183* (K,TAN).

Setaria verticillata (L.) P.Beauv.- Madagascar, Toliara: near SITE 23 Tsimanampesotsa dry forest; Lac Tsimanampesotsa National Park; near the viewpoint closest to the camping site, 24.0884° S, 43.7566° E Alt: 17m, 24 April 2014, *Vorontsova, M.S.*; *Besnard, G.; Razanatsoa, J.; Nanjarisoa, O.P. & Onjalalaina, G.E. 1381* (TAN).

Sirochloa parvifolia (Munro) S.Dransf.- Madagascar, Antsiranana, Diana: SITE 35 Panicum malacotrichum forest gap; Reserve Speciale Manongarivo, Bekolosy forest, 1.5 km N of Ambalifary, 14.0509° S, 48.2940° E Alt: 788m, 12 May 2014, *Vorontsova, M.S.*; *Onjalalaina, G.E. 1462* (TAN).

Sorghum arundinaceum (Desv.) Stapf- Madagascar, Fianarantsoa, Mananjary, Irondro: RN25 from Irondro to Ambolotara, 21.3868° S, 47.9386° E Alt: 82m, 4 November 2011, *Vorontsova, M.S.*; *Hall, R.C.; Besnard, G.; Ralimanana, H.; Randriamboavonjy, T. & Andriantiana, J. 704* (K,TAN).

Sporobolus centrifugus (Trin.) Nees- Madagascar, Fianarantsoa, Fianarantsoa II, Ranomafana: from Fianarantsoa to Ranomafana on RN25, just before the start of Ranomafana National Park, 21.2578° S, 47.3606° E Alt: 1156m, 28 October 2011, *Vorontsova, M.S.*; *Hall, R.C.; Besnard, G.; Ralimanana, H.; Randriamboavonjy, T. & Andriantiana, J. 622* (K,TAN).

Sporobolus perrieri A.Camus- Madagascar, Toliara: SITE 28 Chrysopogon humbertianus; ca 2 km from Tsihombe towards Cape Ste Marie, 25.5843° S, 45.1398° E Alt: 187m, 27 April 2014, *Vorontsova, M.S.*; *Besnard, G.; Razanatsoa, J.; Nanjarisoa, O.P. & Onjalalaina, G.E. 1415* (TAN).

Sporobolus pyramidalis P.Beauv.- Madagascar, Toamasina, Moramanga, Mantadia National Park: Sahanody Riviere, 12 km from Falierana park entrance; roadside at environs of camp 1, 18.8135° S, 48.4301° E Alt: 940m, 8 October 2011, *Vorontsova, M.S.*; *Hall, R.C.; Besnard, G.; Rakotonasolo, F.; Rajaonarison, R. & Rakotondravelo, A. 314* (K,TAN).

Sporobolus virginicus (L.) Kunth- Madagascar, Mahajanga, Antsianitia coast: behind the village beach, 15.5697° S, 46.4156° E Alt: 16m, 14 February 2013, *Vorontsova, M.S.*; *Besnard, G.; Ralimanana, H. & Razanatsoa, J. 926* (K,TAN).

Stenotaphrum oostachyum Baker- Madagascar, Fianarantsoa, Ankaratra Forestry Office: 10km from Ankaratra Forestry Office in Ambatolampy towards Ankaratra forest, 19.3758° S, 47.3553° E Alt: 1575m, 28 February 2013, *Vorontsova, M.S.*; *Besnard, G.; Razanatsoa, J. & Rajaoanarison, R. 1042* (K,TAN).

Styppeiochloa hitchcockii (A.Camus) Cope- Madagascar, Fianarantsoa, Ivato 2: midway from Ivato towards Ambatofinandrahana, 56 km before Ambatofinandrahana, 20.6700° S, 47.1358° E Alt: 1548m, 24 February 2013, *Vorontsova, M.S.*; *Besnard, G.; Razanatsoa, J. & Rajaoanarison, R. 993* (K,TAN).

Themeda quadrivalvis (L.) Kuntze- Madagascar, Antsiranana, Vohemar, Daraina Protected Area office grounds: behind the office building, 13.2064° S, 49.6641° E Alt: 121m, 12 October 2011, *Vorontsova, M.S.*; *Hall, R.C.; Besnard, G.; Rakotonasolo, F.; Rakotoarinivo, M. & Rajaonarison, R. 350* (K,TAN).

Themeda triandra Forssk.- Tanzania, Arusha, Hanang, SITE 54 Hanang pasture: Hanang Mountain, Jerdom valley, Udamaschek village, 4.4975° S, 35.3959° E Alt: 1979m, 15 June 2014, *Vorontsova, M.S.*; *Luke, W.R.Q.; Kimeu, J.M. & Kayombo, C. 1634* (DSM,EA,FTI,K,NHT).

Trachypogon spicatus (L.f.) Kuntze- Madagascar, Fianarantsoa, Ambatofinandrahana, Ankevo: Itremo, 20.6598° S, 46.5794° E Alt: 1682m, 13 March 2012, *Ratovonirina, G.*; *Vorontsova, M.S.; Rakotonasolo, F.; Rakotovao, A. & Rakotonirina, J.N. 192* (K,TAN).

Trichanthecium brazzavillense (Franch.) Zuloaga & Morrone- Madagascar, Fianarantsoa, Ianasana 2: Itremo massif. 2.5km after Ianasana towards centre of the massif, 20.5778° S, 46.5744° E Alt: 1623m, 25 February 2013, *Vorontsova, M.S.*; *Besnard, G.; Razanatsoa, J. & Rajaoanarison, R. 1001* (K,TAN).

Trichanthecium tenellum (Lam.) Zuloaga & Morrone- Tanzania, Rukwa, Mpanda, SITE 55 Uzondo grassland with Aristida

humidicola: Uzondo Plateau S of Uvinza, road from Mpanda, 5.4839° S, 30.5300° E Alt: 1665m, 21 June 2014, *Vorontsova, M.S.*; *Luke, W.R.Q.; Kimeu, J.M. & Kayombo, C. 1653* (K,NHT).

Trycopteryx dregeana Nees- Madagascar, Fianarantsoa, Ambatofinandrahana, Ankevo: Itremo, 20.6599° S, 46.5794° E Alt: 1682m, 13 March 2012, *Ratovonirina, G.*; *Vorontsova, M.S.; Rakotonasolo, F.; Rakotovao, A. & Rakotonirina, J.N. 187* (K,TAN).

Urelytrum agropyroides (Hack.) Hack.- Madagascar, Fianarantsoa, Ambatofinandrahana, Ankevo: Itremo, 20.6599° S, 46.5794° E Alt: 1682m, 13 March 2012, *Ratovonirina, G.*; *Vorontsova, M.S.; Rakotonasolo, F.; Rakotovao, A. & Rakotonirina, J.N. 184* (K,TAN).

Valiha diffusa S.Dransf.- Madagascar, Antsiranana, Diana: SITE 41 Valiha slope; RN6 roadside, 32 km S of Ambanja, just before village Ambodibonga travelling from Ambanja, 13.8563° S, 48.2667° E Alt: 81m, 14 May 2014, *Vorontsova, M.S.*; *Razanatsoa, J.; Nanjarisoa, O.P.; Onjalalaina, G.E. & Rabarivola, M.L. 1487* (TAN).

Valiha sp. - Madagascar, Toliara, SITE 17 BAMBOO FOREST: Isalo National Park, steep slope above Namaza stream, 22.5390° S, 45.3780° E Alt: 784m, 18 December 2013, *Vorontsova, M.S.*; *Linder, H.P.; Nanjarisoa, O.P. & Razanatsoa, J. 1321* (?,TAN).

Viguierella madagascariensis A.Camus- Madagascar, Mahajanga, Station Forestière Marohogo 2: ca 3km on the track from Majunga-Ankarafantsika road, between Station Forestière Marohogo and Andradia, 15.7203° S, 46.4775° E Alt: 39m, 19 February 2013, *Vorontsova, M.S.*; *Besnard, G.; Ralimanana, H. & Razanatsoa, J. 966* (K).

Vulpia bromoides (L.) Gray- Tanzania, Arusha, Hanang, SITE 51 Hanang Protea ridge: Hanang Mountain, Udamaschek, Gabado village territory, 4.4524° S, 35.3875° E Alt: 3079m, 13 June 2014, *Vorontsova, M.S.*; *Luke, W.R.Q.; Kimeu, J.M. & Kayombo, C. 1607* (EA,K,NHT).

Fig. S3 S4 Phylogenetic tree

Chronogram of all species found in the 73 sampled sites based on the plastid regions *rbcL, matK* and *ndhF* and reconstructed using the maximum likelihood criterion as implemented in the programme RAxML and the penalized likelihood approach (see text for details). Number on nodes are bootstrap support values. A. All taxa except Panicoideae. B. Panicoideae.

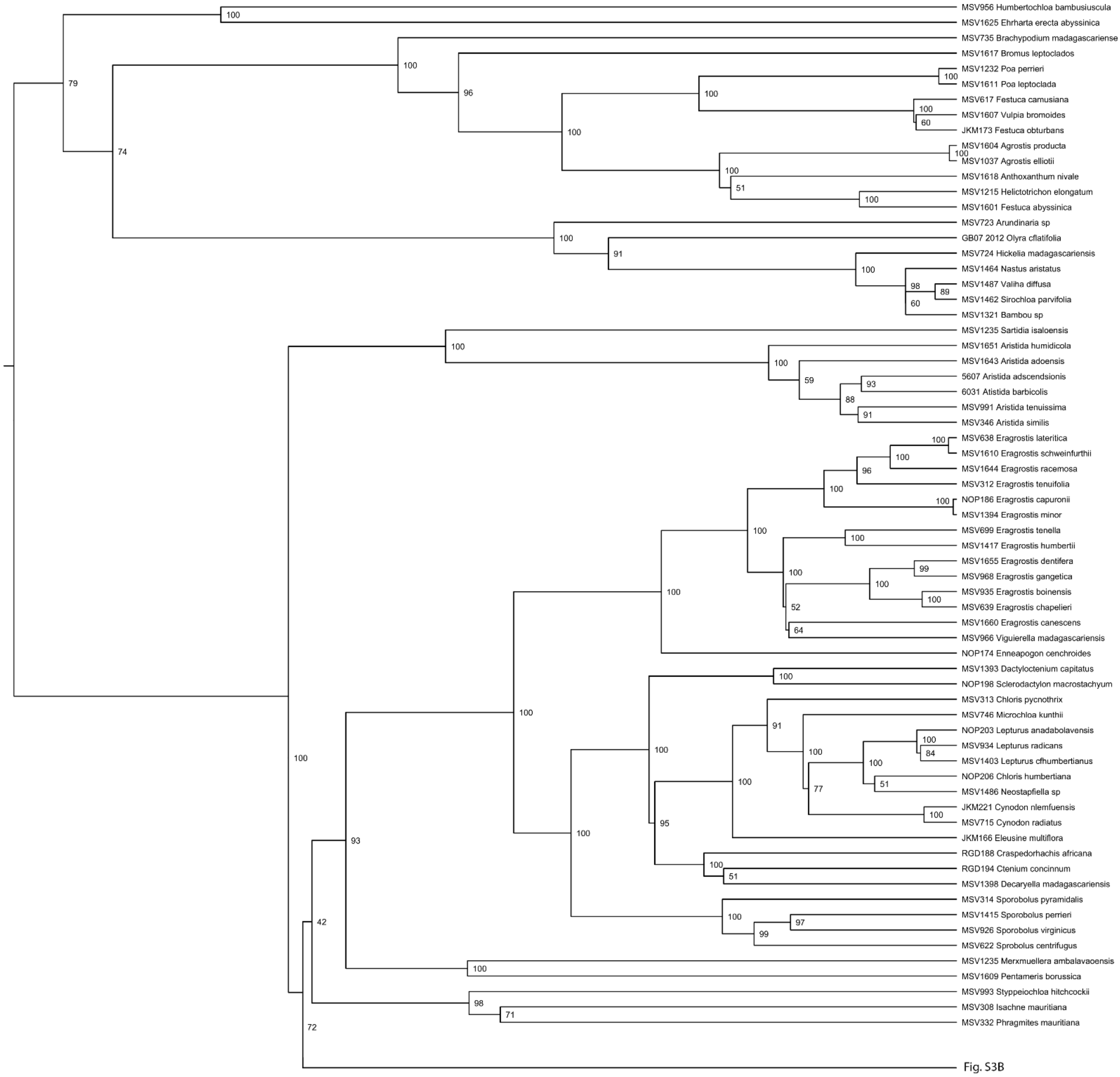

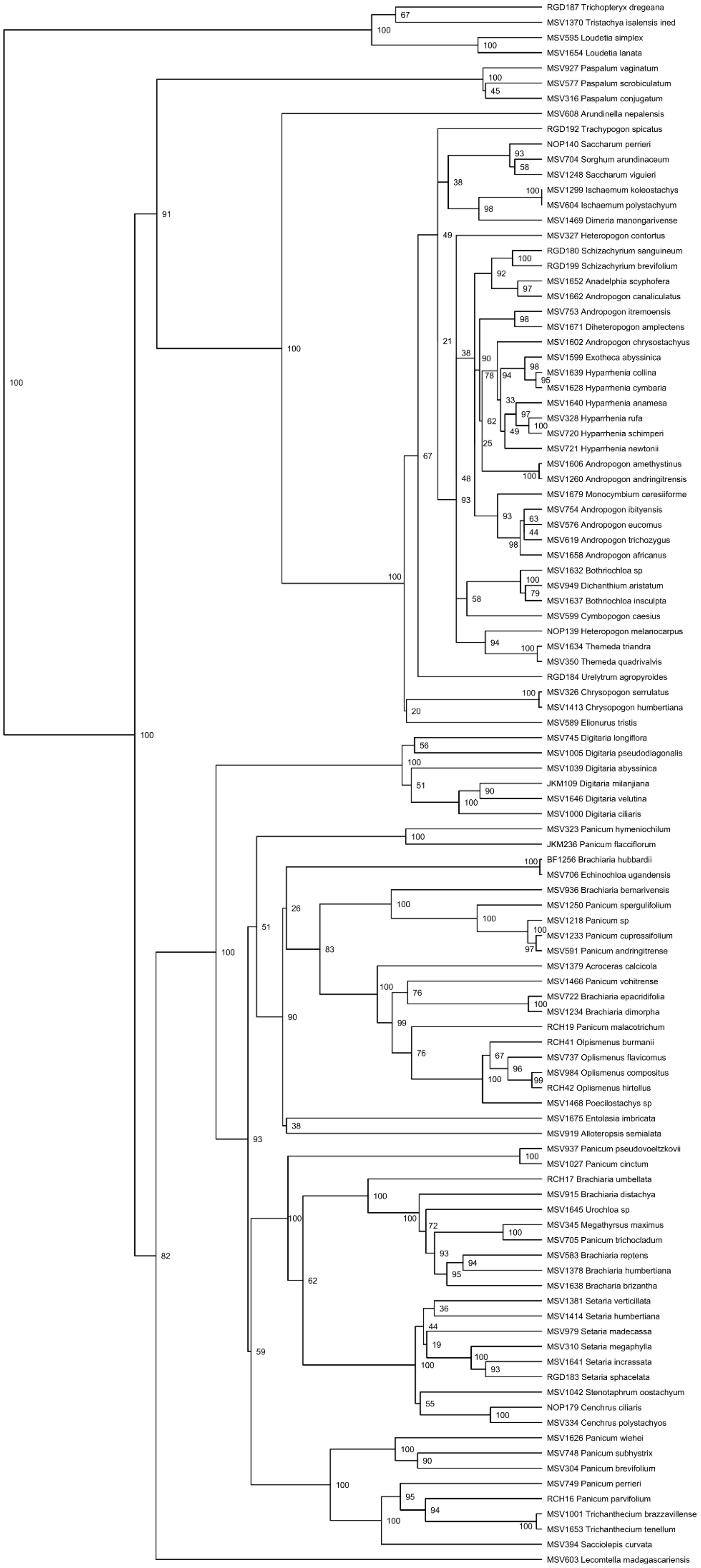
RGD187 Trichopteryx dregeana
MSV1370 Tristachya isalensis ined
MSV595 Loudetia simplex
MSV1654 Loudetia lanata
MSV927 Paspalum vaginatum
MSV577 Paspalum scrobiculatum
MSV316 Paspalum conjugatum
MSV608 Arundinella nepalensis
RGD192 Trachypogon spicatus
NOP140 Saccharum perrieri
MSV704 Sorghum arundinaceum
MSV1248 Saccharum viguieri
MSV1299 Ischaemum koleostachys
MSV604 Ischaemum polystachyum
MSV1469 Dimeria manongarivense
MSV327 Heteropogon contortus
RGD180 Schizachyrium sanguineum
RGD199 Schizachyrium brevifolium
MSV1652 Anadelphia scyphofera
MSV1662 Andropogon canaliculatus
MSV753 Andropogon itremoensis
MSV1671 Diheteropogon amplectens
MSV1602 Andropogon chrysostachyus
MSV1599 Exotheca abyssinica
MSV1639 Hyparrhenia collina
MSV1628 Hyparrhenia cymbaria
MSV1640 Hyparrhenia anamesa
MSV328 Hyparrhenia rufa
MSV720 Hyparrhenia schimperi
MSV721 Hyparrhenia newtonii
MSV1606 Andropogon amethystinus
MSV1260 Andropogon andringitrensis
MSV1679 Monocymbium ceresiiforme
MSV754 Andropogon ibityensis
MSV576 Andropogon eucomus
MSV619 Andropogon trichozygus
MSV1658 Andropogon africanus
MSV1632 Bothriochloa sp
MSV949 Dichanthium aristatum
MSV1637 Bothriochloa insculpta
MSV599 Cymbopogon caesius
NOP139 Heteropogon melanocarpus
MSV1634 Themeda triandra
MSV350 Themeda quadrivalvis
RGD184 Urelytrum agropyroides
MSV326 Chrysopogon serrulatus
MSV1413 Chrysopogon humbertiana
MSV589 Elionurus tristis
MSV745 Digitaria longiflora
MSV1005 Digitaria pseudodiagonalis
MSV1039 Digitaria abyssinica
JKM109 Digitaria milanjiana
MSV1646 Digitaria velutina
MSV1000 Digitaria ciliaris
MSV323 Panicum hymeniochilum
JKM236 Panicum flacciflorum
BF1256 Brachiaria hubbardii
MSV706 Echinochloa ugandensis
MSV936 Brachiaria bemarivensis
MSV1250 Panicum spergulifolium
MSV1218 Panicum sp
MSV1233 Panicum cupressifolium
MSV591 Panicum andringitrense
MSV1379 Acroceras calcicola
MSV1466 Panicum vohitrense
MSV722 Brachiaria epacridifolia
MSV1234 Brachiaria dimorpha
RCH19 Panicum malacotrichum
RCH41 Olpismenus burmanii
MSV737 Oplismenus flavicomus
MSV984 Oplismenus compositus
RCH42 Oplismenus hirtellus
MSV1468 Poecilostachys sp
MSV1675 Entolasia imbricata
MSV919 Alloteropsis semialata
MSV937 Panicum pseudovoeltzkovii
MSV1027 Panicum cinctum
RCH17 Brachiaria umbellata
MSV915 Brachiaria distachya
MSV1645 Urochloa sp
MSV345 Megathyrsus maximus
MSV705 Panicum trichocladum
MSV583 Brachiaria reptens
MSV1378 Brachiaria humbertiana
MSV1638 Bracharia brizantha
MSV1381 Setaria verticillata
MSV1414 Setaria humbertiana
MSV979 Setaria madecassa
MSV310 Setaria megaphylla
MSV1641 Setaria incrassata
RGD183 Setaria sphacelata
MSV1042 Stenotaphrum oostachyum
NOP179 Cenchrus ciliaris
MSV334 Cenchrus polystachyos
MSV1626 Panicum wiehei
MSV748 Panicum subhystrix
MSV304 Panicum brevifolium
MSV749 Panicum perrieri
RCH16 Panicum parvifolium
MSV1001 Trichanthecium brazzavillense
MSV1653 Trichanthecium tenellum
MSV394 Sacciolepis curvata
MSV603 Lecomtella madagascariensis

S4 Checklist of Malagasy grasses

List of 541 species of Poaceae in Madagascar, not including Comores or Mascarenes. 217 species endemic to Madagascar are marked with E.

This list is based on unpublished work by Emmet Judziewicz on non-bambusoid grasses and unpublished work on bamboos by Soejatmi Dransfield, revised following field and herbarium work partially published in Vorontsova 2013, Vorontsova et al. 2013, Vorontsova 2014, Vorontsova & Rakotoarisoa 2014, Vorontsova et al. 2014, and Vorontsova et al. in press. Generic affiliations follow Kellogg (2015); where the correct combination is not available the accepted generic name is followed by the outdated name in brackets. This article does not intend to publish any new names. Undescribed species are not included.

1. Acrachne perrieri E
2. Acroceras boivinii E
3. Acroceras calcicola E
4. Acroceras ivohibense E
5. Acroceras malacotrichum E
6. Acroceras parvulum E
7. Acroceras tenuicaule E
8. Adenochloa hymeniochila
9. Agrostis decaryana E
10. Agrostis elliotii E
11. Agrostis humbertii E
12. Agrostis marojejyensis E
13. Agrostis perennans
14. Agrostis tsaratananensis E
15. Agrostis tsiafajavonensis E
16. Agrostis tsitondroinensis E
17. Alloteropsis cimicina
18. Alloteropsis paniculata
19. Alloteropsis semialata
20. Andropogon andringitrense E
21. Andropogon eucomus
22. Andropogon huillensis
23. Andropogon ibityensis E
24. Andropogon imerinensis E
25. Andropogon itremoensis E
26. Andropogon ivohibensis E
27. Andropogon trichozygus E
28. Andropogon tsaratananensis E
29. Anthoxanthum madagascariense E
30. Anthoxanthum odoratum
31. Aristida adscensionis
32. Aristida ambongensis E
33. Aristida barbicollis
34. Aristida congesta
35. Aristida cumingiana
36. Aristida junciformis
37. Aristida rufescens E
38. Aristida similis E
39. Aristida tenuissima E
40. Arthraxon antsirabensis E
41. Arthraxon lancifolius
42. Arundinaria ambositrensis E
43. Arundinaria humbertii E
44. Arundinaria ibityensis E
45. Arundinaria madagascariensis E
46. Arundinaria marojejyensis E
47. Arundinaria perrieri E
48. Arundinella nepalensis
49. Axonopus compressus
50. Bambusa multiplex
51. Bambusa vulgaris
52. Bothriochloa bladhii
53. Bothriochloa insculpta
54. Bothriochloa pertusa
55. Brachiaria antsirabensis E
56. Brachiaria arrecta
57. Brachiaria bemarivensis E
58. Brachiaria brizantha
59. Brachiaria comorensis
60. Brachiaria deflexa
61. Brachiaria dimorpha E
62. Brachiaria distachya
63. Brachiaria epacridifolia E
64. Brachiaria fragrans E
65. Brachiaria fruticulosa E
66. Brachiaria hubbardii
67. Brachiaria humbertiana E
68. Brachiaria jubata
69. Brachiaria leandriana E
70. Brachiaria mutica
71. Brachiaria nana E
72. Brachiaria perrieri E
73. Brachiaria pseudodichotoma E
74. Brachiaria ramosa

75. Brachiaria ruziziensis
76. Brachiaria subquadripara
77. Brachiaria subrostrata E
78. Brachiaria tsiafajavonensis E
79. Brachiaria umbellata
80. Brachypodium flexum
81. Brachypodium humbertianum E
82. Brachypodium madagascarensis E
83. Brachypodium perrieri E
84. Briza minor
85. Bromus andringitrensis E
86. Bromus pectinatus
87. Calamagrostis emirnensis E
88. Cathariostachys capitata E
89. Cathariostachys madagascariensis E
90. Cenchrus (Pennisetum pseudotriticoides)
91. Cenchrus (Pennisetum villosum)
92. Cenchrus americanus
93. Cenchrus biflorus
94. Cenchrus ciliaris
95. Cenchrus clandestinus
96. Cenchrus echinatus
97. Cenchrus hohenackeri
98. Cenchrus melanostachyus E
99. Cenchrus pedicellatus
100. Cenchrus polystachios
101. Cenchrus purpureus
102. Cephalostachyum chevalieri E
103. Cephalostachyum perrieri E
104. Cephalostachyum viguieri E
105. Chloris barbata
106. Chloris filiformis
107. Chloris gayana
108. Chloris humbertiana E
109. Chloris pycnothrix
110. Chloris roxburghiana
111. Chloris virgata
112. Chrysopogon humbertianus E
113. Chrysopogon serrulatus
114. Chrysopogon zizanioides
115. Coelachne africana
116. Coelachne simpliciuscula
117. Coix lacryma-jobi
118. Craspedorhachis africana
119. Craspedorhachis rhodesiana
120. Crypsis schoenoides
121. Ctenium concinnum
122. Cymbopogon caesius
123. Cymbopogon citratus
124. Cymbopogon flexuosus
125. Cymbopogon giganteus
126. Cymbopogon martinii
127. Cymbopogon nardus
128. Cymbopogon pruinosus
129. Cynodon coursii E
130. Cynodon dactylon
131. Cynodon plectostachyus
132. Cynodon radiatus
133. Cynodon transvaalensis
134. Cyphochlaena madagascariensis
135. Cyphochlaena sclerioides
136. Cyrtococcum bosseri E
137. Cyrtococcum deltoideum E
138. Cyrtococcum humbertianum E
139. Cyrtococcum multinode
140. Cyrtococcum nossibeense E
141. Cyrtococcum oxyphyllum
142. Cyrtococcum tamatavense E
143. Cyrtococcum trigonum
144. Dactyloctenium aegyptium
145. Dactyloctenium australe
146. Dactyloctenium capitatum E
147. Dactyloctenium ctenoides
148. Dactyloctenium geminatum
149. Dactyloctenium giganteum
150. Dactyloctenium pilosum
151. Daknopholis boivinii
152. Decaryella madagascariensis E
153. Decaryochloa diadelpha E
154. Dendrocalamus asper
155. Dendrocalamus giganteus
156. Dichanthium annulatum
157. Dichanthium aristatum
158. Diectomis fastigiatus
159. Digitaria abyssinica
160. Digitaria ankaratrensis E
161. Digitaria argyrograpta
162. Digitaria argyrotricha
163. Digitaria atrofusca
164. Digitaria bicornis
165. Digitaria ciliaris
166. Digitaria debilis
167. Digitaria didactyla
168. Digitaria fulva E
169. Digitaria fuscescens
170. Digitaria gazensis

171. Digitaria horizontalis
172. Digitaria humbertii E
173. Digitaria longiflora
174. Digitaria madagascariensis E
175. Digitaria manongarivensis E
176. Digitaria nuda
177. Digitaria perrieri E
178. Digitaria perrottetii
179. Digitaria planiculmis E
180. Digitaria psammophila E
181. Digitaria pseudodiagonalis
182. Digitaria radicosa
183. Digitaria setifolia
184. Digitaria setigera
185. Digitaria thouaresiana
186. Digitaria tricholaenoides
187. Digitaria velutina
188. Digitaria violascens
189. Dimeria madagascariensis E
190. Dimeria manongarivensis E
191. Dimeria perrieri E
192. Dinebra caerulescens
193. Dinebra panicea
194. Dinebra perrieri E
195. Dinebra retroflexa
196. Dinebra squarrosa
197. Diplachne fusca
198. Echinochloa colona
199. Echinochloa crusgalli
200. Echinochloa pyramidalis
201. Echinochloa stagnina
202. Echinolaena egregia E
203. Echinolaena humbertiana E
204. Echinolaena madagascariensis E
205. Ehrharta stipoides
206. Eleusine africana
207. Eleusine coracana
208. Eleusine indica
209. Elionurus tristis
210. Enneapogon cenchroides
211. Enteropgon prieurii
212. Enteropogon monostachyos
213. Enteropogon sechellensis
214. Eragrostis aethiopica
215. Eragrostis ambohibengensis E
216. Eragrostis ambositrensis E
217. Eragrostis ambrensis E
218. Eragrostis aspera
219. Eragrostis atrovirens
220. Eragrostis bemarivensis
221. Eragrostis betsileensis E
222. Eragrostis boinensis E
223. Eragrostis capensis
224. Eragrostis capuronii E
225. Eragrostis chabouisii E
226. Eragrostis chapelieri E
227. Eragrostis cilianensis
228. Eragrostis ciliaris
229. Eragrostis curvula
230. Eragrostis cylindriflora
231. Eragrostis gangetica
232. Eragrostis gummiflua
233. Eragrostis hildebrandtii E
234. Eragrostis humbertii E
235. Eragrostis japonica
236. Eragrostis lateritica E
237. Eragrostis macilenta
238. Eragrostis majungensis E
239. Eragrostis mandrarensis E
240. Eragrostis minor
241. Eragrostis multicaulis
242. Eragrostis patula
243. Eragrostis perrieri E
244. Eragrostis pilosa
245. Eragrostis plana
246. Eragrostis racemosa
247. Eragrostis sambiranensis E
248. Eragrostis sarmentosa
249. Eragrostis stolonifera E
250. Eragrostis subaequiglumis
251. Eragrostis tef
252. Eragrostis tenella
253. Eragrostis tenuifolia
254. Eragrostis unioloides
255. Eragrostis viguieri E
256. Eriochloa fatmensis
257. Eriochloa meyeriana
258. Eriochloa parvispiculata
259. Eriochloa procera
260. Eriochloa stapfiana
261. Eriochloa subulifera
262. Euclasta condylotricha
263. Eulalia aurea
264. Eulalia villosa
265. Festuca (Pseudobromus breviligulatus) E

266. Festuca (Pseudobromus tenuifolius) E
267. Festuca ambilobensis E
268. Festuca brevipaleata E
269. Festuca camusiana E
270. Festuca humbertii E
271. Festuca perrieri E
272. Gigantochloa pseudoarundinacea
273. Hackelochloa granularis
274. Halopyrum mucronatum
275. Helictotrichon elongatum
276. Helictotrichon milanjianum
277. Hemarthria altissima
278. Heteropholis benoistii E
279. Heteropogon contortus
280. Heteropogon melanocarpus
281. Hickelia alaotrensis E
282. Hickelia madagascariensis E
283. Hickelia perrieri E
284. Hitchcockella baronii E
285. Humbertochloa bambusiuscula E
286. Hyparrhenia cymbaria
287. Hyparrhenia diplandra
288. Hyparrhenia filipendula
289. Hyparrhenia hirta
290. Hyparrhenia newtonii
291. Hyparrhenia nyassae
292. Hyparrhenia rufa
293. Hyparrhenia schimperi
294. Hyparrhenia variabilis
295. Hyperthelia dissoluta
296. Imperata cylindrica
297. Isachne ciliaris
298. Isachne hirtissima E
299. Isachne humbertiana E
300. Isachne humicola E
301. Isachne mauritiana
302. Isachne muscicola E
303. Ischaemum ciliare
304. Ischaemum heterotrichum
305. Ischaemum koleostachys
306. Ischaemum polystachyum
307. Ischaemum rugosum
308. Lecomtella madagascariensis E
309. Leersia hexandra
310. Leersia perrieri
311. Leptaspis zeylanica
312. Leptocarydion vulpiastrum
313. Lepturus anadabolavensis E
314. Lepturus androyensis E
315. Lepturus boinensis E
316. Lepturus humbertianus E
317. Lepturus perrieri E
318. Lepturus radicans
319. Lepturus repens
320. Loudetia filifolia
321. Loudetia simplex
322. Maltebrunia leersioides E
323. Maltebrunia maroana E
324. Megastachya madagascariensis E
325. Megastachya mucronata
326. Melinis minutiflora
327. Melinis nerviglumis
328. Melinis repens
329. Merxmuellera ambalavaoensis E
330. Merxmuellera macowanii E
331. Merxmuellera tsaratananensis E
332. Microchloa kunthii
333. Microstegium nudum
334. Moorochloa eruciformis
335. Nastus ambrensis E
336. Nastus aristatus E
337. Nastus decaryanus E
338. Nastus elongatus E
339. Nastus emirnensis E
340. Nastus humbertianus E
341. Nastus lokohensis E
342. Nastus madagascariensis E
343. Nastus manongarivensis E
344. Nastus perrieri E
345. Nastus tsaratananensis E
346. Neostapfiella chloridiantha E
347. Neostapfiella humbertiana E
348. Neostapfiella perrieri E
349. Neyraudia arundinacea
350. Olyra latifolia
351. Oplismenus burmannii
352. Oplismenus compositus
353. Oplismenus flavicomus E
354. Oplismenus hirtellus
355. Oplismenus undulatifolius
356. Oryza longistaminata

357. Oryza punctata
358. Oryza sativa
359. Oxyrhachis gracillima
360. Panicum ambositrense E
361. Panicum andringitrense E
362. Panicum ankarense E
363. Panicum bathiei E
364. Panicum brevifolium
365. Panicum capuronii E
366. Panicum cinctum E
367. Panicum crystallinum E
368. Panicum cupressifolium E
369. Panicum danguyi E
370. Panicum dregeanum E
371. Panicum flacourtii E
372. Panicum ibityense E
373. Panicum inconspicuum E
374. Panicum leptolomoides E
375. Panicum luridum E
376. Panicum manongarivense E
377. Panicum mitopus
378. Panicum neobathiei E
379. Panicum neohumbertii E
380. Panicum neoperrieri E
381. Panicum novemnerve
382. Panicum palackyanum E
383. Panicum perrieri E
384. Panicum pleianthum E
385. Panicum spergulifolium E
386. Panicum subalbidum
387. Panicum subhystrix E
388. Panicum trichocladum
389. Panicum voeltzkowii E
390. Panicum vohitrense E
391. Panicum walense
392. Paratheria prostrata
393. Paspalidium ankarense E
394. Paspalidium flavidum
395. Paspalidium geminatum
396. Paspalum conjugatum
397. Paspalum dilatatum
398. Paspalum glumaceum
399. Paspalum notatum
400. Paspalum nutans
401. Paspalum paniculatum
402. Paspalum scrobiculatum
403. Paspalum urvillei
404. Paspalum vaginatum
405. Paspalum virgatum
406. Pentameris andringitrensis E
407. Pentameris humbertii E
408. Pentameris natalensis E
409. Perotis hildebrandtii
410. Perotis humbertii E
411. Perotis indica
412. Perotis patens
413. Perrierbambus madagascariensis E
414. Perrierbambus tsarasaotrensis E
415. Phragmites mauritianus
416. Phyllostachys aurea
417. Poa ankaratrensis E
418. Poa annua
419. Poa madecassa E
420. Poa perrieri E
421. Poecilostachys ambositrensis E
422. Poecilostachys bakeri E
423. Poecilostachys confertiflora E
424. Poecilostachys geminatus E
425. Poecilostachys gougerotiana E
426. Poecilostachys hildebrandtii E
427. Poecilostachys humbertii E
428. Poecilostachys mainborondroensis E
429. Poecilostachys manongarivensis E
430. Poecilostachys marojejyensis E
431. Poecilostachys mollis E
432. Poecilostachys muscicola E
433. Poecilostachys tsaratananensis E
434. Poecilostachys viguieri E
435. Pogonarthria squarrosa
436. Pogonatherum paniceum
437. Pseudechinolaena camusiana E
438. Pseudechinolaena madagascariensis E
439. Pseudechinolaena moratii E
440. Pseudechinolaena perrieri E
441. Pseudechinolaena polystachya
442. Rhytachne rottboellioides
443. Rottboellia cochinchinensis
444. Saccharum hildebrandtii E
445. Saccharum officinarum

446. Saccharum perrieri E
447. Saccharum viguieri E
448. Sacciolepis africana
449. Sacciolepis chevalieri E
450. Sacciolepis curvata
451. Sacciolepis indica
452. Sacciolepis micrococca
453. Sacciolepis myosuroides
454. Sacciolepis viguieri E
455. Sartidia isaloensis E
456. Sartidia perrieri E
457. Schizachyrium brevifolium
458. Schizachyrium exile
459. Schizachyrium platyphyllum
460. Schizachyrium sanguineum
461. Schizostachyum bosseri
462. Schizostachyum perrieri
463. Schoenefeldia gracilis
464. Sclerodactylon macrostachyum
465. Setaria barbata
466. Setaria bathiei E
467. Setaria bosseri E
468. Setaria fiherenensis E
469. Setaria humbertiana E
470. Setaria madecassa E
471. Setaria megaphylla
472. Setaria parviflora
473. Setaria perrieri E
474. Setaria pumila
475. Setaria sagittifolia
476. Setaria scabrifolia
477. Setaria scottii E
478. Setaria sphacelata
479. Setaria taolanensis E
480. Setaria vatkeana E
481. Setaria verticillata
482. Sirochloa parvifolia E
483. Sorghastrum incompletum
484. Sorghum arundinaceum
485. Sorghum bicolor
486. Sporobolus africanus
487. Sporobolus bosseri E
488. Sporobolus centrifugus
489. Sporobolus coromandelianus
490. Sporobolus diandrus
491. Sporobolus elatior E
492. Sporobolus festivus
493. Sporobolus halophilus E
494. Sporobolus indicus
495. Sporobolus micranthus
496. Sporobolus paniculatus
497. Sporobolus perrieri E
498. Sporobolus piliferus
499. Sporobolus pyramidalis
500. Sporobolus rigidifolius
501. Sporobolus stapfianus
502. Sporobolus subtilis
503. Sporobolus subulatus
504. Sporobolus tenuissimus
505. Sporobolus virginicus
506. Stenotaphrum dimidiatum
507. Stenotaphrum micranthum
508. Stenotaphrum oostachyum E
509. Stenotaphrum unilaterale E
510. Styppeiochloa hitchcockii E
511. Themeda quadrivalvis
512. Themeda triandra
513. Thuarea involuta
514. Thuarea perrieri E
515. Trachypogon spicatus
516. Tragus berteronianus
517. Tragus mongolorum
518. Trichanthecium brazzavillense
519. Trichanthecium parvifolium
520. Tricholaena monachne
521. Trichopteryx dregeana
522. Tripogon minimus
523. Tripsacum andersonii
524. Tristachya (Isalus isalensis) E
525. Tristachya betsileensis E
526. Tristachya humbertii E
527. Triticum aestivum
528. Urelytrum agropyroides
529. Urochloa glumaris
530. Urochloa maxima
531. Urochloa mosambicensis
532. Urochloa panicoides
533. Urochloa plantaginea
534. Urochloa reptans
535. Valiha diffusa E
536. Valiha perrieri E
537. Viguierella madagascariensis E
538. Vulpia myuros
539. Yvesia madagascariensis E
540. Zea mays
541. Zoysia matrella

Besnard, G., Christin, P. A., Malé, P. J. G., Lhuillier, E., Lauzeral, C., Coissac, E., & Vorontsova, M. S. (2014). From museums to genomics: old herbarium specimens shed light on a C3 to C4 transition. Journal of Experimental Botany 65 (22): 6711-6721.

Kellogg, E. 2015. Families and Genera of Flowering Plants, volume XIII: Poaceae. Edited by K. Kubitzki. Springer.

Vorontsova, M.S. 2013. Variable morphology of the Madagascar endemic Aristida tenuissima (Poaceae: Aristidoideae) and the absence of Stipa (Poaceae: Pooideae, Stipeae) from Madagascar. Phytotaxa 92: 55-58.

Vorontsova, M.S., G. Ratovonirina & T. Randriamboavonjy. 2013. Revision of Andropogon and Diectomis (Poaceae: Sacchareae) in Madagascar and the new Andropogon itremoensis from the Itremo Massif. Kew Bulletin 68(2): 193-207.

Vorontsova, M.S. 2014. Two new species of Panicum sensu lato (Poaceae: Panicoideae) from Madagascar. Kew Bulletin 69: 9511. DOI 10.1007/S12225-014-9511-3

Vorontsova, M.S., S. E. Rakotoarisoa. 2014. Endemic non-bambusoid genera of grasses (Poaceae) in Madagascar: review of current knowledge. Malagasy Nature 8: 14-34.

Vorontsova, M.S., O. P. Nanjarisoa & G. Besnard. 2014. Three new grass records for Madagascar. In: M. Callmander & P. Phillipson (eds.), Notes on the flora of Madagascar 36. Candollea 69(1): 85-87.

Vorontsova, M.S., T. Haevermans, A. Haevermans, J. Razanatsoa & G. Besnard. The genus Sartidia (Poaceae: Aristidoideae) in Madagascar. Systematic Botany, in press.
